# Pantothenic Acid Derivatives Modulate Oxidative Stress and Hepatic Fibrosis in Bile Duct Ligation-Induced Cholestatic Liver Injury

**DOI:** 10.3390/pathophysiology33020032

**Published:** 2026-05-13

**Authors:** Dmitry S. Semenovich, Polina A. Abramicheva, Ljubava D. Zorova, Andrey V. Elchaninov, Maria A. Kozlova, David A. Areshidze, Nadezda V. Andrianova, Nina P. Kanunnikova, Andrey G. Moiseenok, Irina B. Pevzner, Egor Y. Plotnikov, Dmitry B. Zorov

**Affiliations:** 1 A.N. Belozersky Institute of Physico-Chemical Biology, M.V. Lomonosov Moscow State University, 119992 Moscow, Russia; abramicheva.polina@belozersky.msu.ru (P.A.A.); ljuzor@belozersky.msu.ru (L.D.Z.); pevzner_ib@belozersky.msu.ru (I.B.P.); zorov@belozersky.msu.ru (D.B.Z.); 2 V.I. Kulakov National Medical Research Center of Obstetrics, Gynecology, and Perinatology, 117997 Moscow, Russia; 3 Avtsyn Research Institute of Human Morphology, Federal State Budgetary Scientific Institution “Petrovsky National Research Centre of Surgery”, 117418 Moscow, Russia; elchandrey@yandex.ru (A.V.E.); kma-morph@mail.ru (M.A.K.); labcelpat@mail.ru (D.A.A.); 4 Department of Technology, Physiology and Food Hygiene, Yanka Kupala State University of Grodno, 230022 Grodno, Belarus; n.kanunnikova@grsu.by; 5 Institute of Biochemistry of Biologically Active Compounds, 230030 Grodno, Belarus; andrey.moiseenok@tut.by

**Keywords:** common bile duct ligation, glutathione system, inflammation, liver fibrosis, lipid peroxidation, panthenol, pantethine, hopantenic acid, obstructive cholestasis, oxidative stress, mitochondria

## Abstract

Background/Objectives: Inflammation and oxidative stress are key factors contributing to the initiation and progression of liver fibrosis in chronic obstructive cholestasis. Pantothenic acid (PA) and some of its derivatives have been reported to exhibit moderate anti-inflammatory, antioxidant, and regenerative effects. This study aimed to evaluate the redox-modulating effects of PA derivatives—panthenol (PL), pantethine (PT), and hopantenic acid (HPA) in a rat model of chronic obstructive cholestasis induced by common bile duct ligation (BDL). Methods: Macroscopic, histological, and ultrastructural alterations in the liver were assessed, along with molecular markers of oxidative stress, inflammation, and parameters of the glutathione (GSH) system. Results: BDL-induced liver injury was associated with enhanced lipid peroxidation, mitochondrial structural alterations, depletion of GSH, increased levels of protein S-glutathionylation (PSSG), and elevated thiobarbituric acid-reactive substances in mitochondria. Treatment with PL and, to a lesser extent, PT was associated with attenuation of hepatocellular ultrastructural damage, reduced bile duct hyperplasia, decreased inflammatory and necrotic changes, and moderate improvement in fibrosis-related parameters. In contrast, HPA (a PA antagonist) did not demonstrate hepatoprotective effects and it was associated with more pronounced liver injury. Conclusions: Chronic BDL is accompanied by suppression of glutathione redox capacity and enhanced oxidative stress. PL and PT, but not HPA, were associated with reduced levels of protein S-glutathionylation and partial restoration of redox balance. The protective effects of PL and PT may contribute to their antifibrotic activity, potentially through direct antioxidant capacity or redox-modulating mechanisms associated with the GSH system.

## 1. Introduction

Cholestatic liver disease is a condition that causes liver cell damage and fibrosis due to impaired secretion and/or impaired bile out flow through the bile ducts [1]. This leads to increased accumulation of potentially toxic bile acids (BAs) in the liver and blood serum, accompanied by the development of jaundice [2]. In response to the damaging effects of BAs, cholangiocytes proliferate and activate hepatic stellate cells (HSCs) and fibroblasts, leading to the proliferation of extracellular matrix (ECM), which yield fibrosis [3]. Although the disease may progress slowly, manifesting itself over a long period of time as only minor liver dysfunction, it can lead to cirrhosis or liver failure requiring liver transplantation [4].

Oxidative stress is a key factor in initiating tissue fibrosis associated with chronic cholestasis [5]. Mitochondria are a target for BAs-induced cytotoxicity [6]. BAs are known to cause dissipation of mitochondrial membrane potential, mitochondrial swelling, decreased ATP synthesis, high production of reactive oxygen species (ROS), and initiation of apoptosis [7].

It has previously been shown that pantothenic acid (PA, vitamin B5) had an antifibrotic effect in rats with chronic toxic liver damage caused by tetrachloromethane and in mice with thioacetamide-induced liver fibrosis, reducing collagen deposition and proinflammatory markers, as well as attenuating HSCs activation [8,9].

Some PA derivatives also have a pharmacological potential for the treatment of chronic liver diseases. In particular, panthenol (PL), an alcohol derivative of PA with anti-inflammatory, anti-apoptotic, and antioxidant effects, has been successfully used in models of acute toxic injury, sepsis and subtotal liver ischemia–reperfusion [10,11,12,13]. PL is a precursor for the biosynthesis of coenzyme A (CoA) [14], which, as has recently been shown, exhibits antioxidant activity and participates in redox reactions with proteins, protecting their free SH-groups from oxidation by ROS [15]. It is also known that PA and PL are capable of enhancing glutathione biosynthesis in lymphoblast cells [16]. We have previously shown that PL protects mitochondria from oxidative stress in vitro by reducing the level of carbonyl products of lipid peroxidation and stabilizing the thiol-disulfide redox status [17].

Another unique derivative of PA is pantethine (PT), a disulfide of pantetheine, which is part of the CoA molecule. In its phosphorylated form (4′-phosphopantetheine), it is part of acyl-carrying protein (ACP), ensuring key reactions in the metabolism of fatty acids and thioesters [18]. Pantetheine is a source of endogenous pantothenate and cysteamine, which are formed in a reaction catalyzed by pantetheinase (vanin) [19]. PT is known to have antioxidant and hypolipidemic effects and to protect the liver from fatty degeneration and toxic damage caused by tetrachloromethane [20,21]. Some PT metabolites, in particular cysteamine and taurine, have a hepatoprotective effect due to their antifibrotic, anti-inflammatory, and antioxidant effects [22,23].

Among PA derivatives, there are compounds with antagonistic effects, in particular hopantenic acid (HPA). HPA (or N-pantoyl-GABA) is a homologue of PA (N-pantoyl-β-alanine) containing an additional methylene group in the composition of the GABA molecule fragment. HPA can act as a competitive inhibitor of pantothenate kinase [24], which significantly distinguishes HPA from PL and PT in terms of metabolism. It has recently been established that HPA exhibits potential anti-inflammatory (modulation of prostaglandin metabolism) and antitumor effects (modulation of steroid metabolism, increased genomic DNA stability) [25]. Thus, the question of the effectiveness of various structural derivatives of PA for antifibrotic therapy in chronic liver diseases remains open.

We have suggested that the mechanism of the antifibrotic action of PL and PT may be in their ability to modulate redox-dependent transformations in different cellular compartments and their sensitivity to oxidative stress, which may be accompanied by the activation of protective antioxidant systems, a reduction in inflammation, and profibrogenic molecular markers in the liver. The aim of the present study was to evaluate the hepatoprotective effects of pantothenic acid derivatives (PL, PT, and HPA), with a focus on inflammation, oxidative stress, and parameters of the glutathione system in liver fibrosis in rats with chronic obstructive cholestasis.

## 2. Materials and Methods

### 2.1. Animals

Male outbred Wistar rats aged 7–9 months with an initial body weight of 430–500 g (*n* = 30) were used. Rats were kept in accordance with protocols approved by the Animal Care Ethics Committee of the A.N. Belozersky Research Institute of Physico-Chemical Biology, Lomonosov Moscow State University (protocol 016-1/02/2025 from 9 June 2025) and according to the ARRIVE guidelines. Rats in all experimental groups had unlimited access to drinking water. Throughout the study, rats received pelleted food from Laboratorkorm (Laboratorkorm, Moscow, Russia) with an energetic value of 300 kcal/100 g. Rats were kept under controlled temperatures ranging from 18 °C to 22 °C and had a 12/12-h light cycle.

### 2.2. Experimental Model of Bile Duct Ligation and Treatment Protocol

Before surgery, rats (*n* = 30) were fasted for 14–16 h with free access to drinking water. Under anesthesia with chloral hydrate (300 mg/kg, intraperitoneally), a midline laparotomy was performed, and the liver was accessed. For BDL modeling (*n* = 25), the common bile duct was isolated, and two ligatures were applied with 3-0 silk suture. The duct was divided between the ligatures. In sham-operated rats (*n* = 5), the common bile duct was isolated but not ligated or transected. The surgical site was sutured and treated with 1% brilliant green solution. To prevent postoperative infection, rats were administered the antibiotic cefazolin (20 mg/kg, single dose, intramuscularly). Sutures were removed 7–10 days after surgery. During the first three weeks after surgery, 5 out of 25 rats with BDL died (20% mortality).

**Figure 1:**
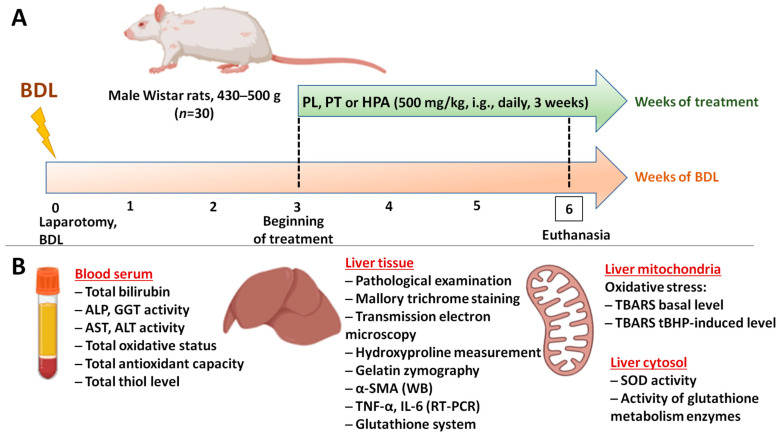
Experimental design. (**A**) Scheme of the execution of BDL model and treatment with PA derivatives. (**B**) Analyzed parameters in serum, tissue, and subcellular liver fractions of rats. Created in BioRender. Semenovich, D.S. (2026). https://BioRender.com/6m665ot.

Three weeks after surgery, all surviving BDL rats (*n* = 20) were randomly allocated into experimental groups receiving PA derivatives (PL, PT, and HPA) at an equivalent dose of 500 mg/kg, intragastrically, daily from 3 to 6 weeks after obstruction (Figure 1A). Sham-operated and untreated BDL control rats received an equal volume of distilled water.

Accordingly, the following experimental groups were established:

1—Sham (*n* = 5);

2—BDL (*n* = 5);

3—BDL + PL (*n* = 5);

4—BDL + PT (*n* = 5);

5—BDL + HPA (*n* = 5).

No mortality was observed during weeks 3 to 6 of treatment with PA derivatives (100% survival). The Supplementary Materials (see Table S1) provide the number of rats placed in each cohort and the number allocated for the specific research protocols.

At week 6 of the experiment, the animals were euthanized under chloral hydrate anesthesia (300 mg/kg intraperitoneally) and blood and liver samples were then collected for biochemical and morphological analyses (Figure 1B).

### 2.3. Biochemical Analysis of Blood Serum

On the day of euthanasia, blood was collected in tubes containing a clot activator and separating gel (MiniMed, Suponevo, Russia). To obtain serum, blood samples were allowed to clot at room temperature in the dark for 30 min and then centrifuged at 1500× *g* for 10 min. The separated serum was collected and stored at −70 °C and later used for biochemical analysis.

The assessment of cholestatic liver damage was based on changes in the concentration of total bilirubin and the activity of indicator liver enzymes in the blood serum.

Total bilirubin concentration was determined spectrophotometrically at 550 nm using the Walters-Gerarde method with a commercial reagent kit (Olvex Diagnosticum, St. Petersburg, Russia) in accordance with the manufacturer’s instructions.

Alkaline phosphatase (ALP) activity was measured spectrophotometrically at 405 nm using p-nitrophenyl phosphate as a substrate, while γ-glutamyltransferase (GGT) activity was determined at 405 nm using L-γ-glutamyl-3-carboxy-4-nitroanilide as a substrate in the presence of glycylglycine. The activities of aspartate aminotransferase (AST) and alanine aminotransferase (ALT) in serum were determined spectrophotometrically at 546 nm using the Reitman–Frankel method. All measurements were performed using commercial reagent kits from Olvex Diagnosticum (St. Petersburg, Russia) following the manufacturer’s protocol.

The TOS of serum was determined spectrophotometrically based on the formation of a colored pink complex with N,N-dimethyl-p-phenylenediamine (DMPD) at 505 nm [26]. The calibration curve was constructed using a solution of ferric chloride as an oxidizing agent in the concentration range of 20–200 μM [27].

The TAC of serum was evaluated spectrophotometrically by monitoring the reduction of the ABTS cation radical at 660 nm [28]. TAC values were expressed in μM GSH equivalents, based on a calibration curve prepared with GSH (100–1000 μM).

TSH concentration in serum was determined spectrophotometrically according to the Ellman method, which measures the reaction product of free thiol groups with Ellman’s reagent (DTNB) at 412 nm [29]. The concentration of total thiols was calculated from a calibration curve prepared using standard GSH solutions (100–1000 μM).

Changes in absorbance were monitored using Ekros PE-5400UV spectrophotometer (Ekrokhim, St. Petersburg, Russia).

### 2.4. Evaluation of Changes in Body Weight of Rats, Macro- and Microscopic Examination of the Liver

The body weight of each rat was recorded throughout the study. In week 6, the animals were euthanized and necropsied. To document macroscopic changes, the liver was weighed and photographed. Liver weight was expressed as a percentage of the rat’s body weight.

Histological examination of the liver was performed to assess changes and monitor the development of fibrosis. Liver tissue was fixed in 10% buffered formalin and embedded in paraffin using the established method. Paraffin sections (5 µm thick) were stained with Mallory trichrome using a BioVitrum kit (BioVitrum, St. Petersburg, Russia) according to the manufacturer’s instructions. Stained sections were examined under an AxioScope A1 microscope (Carl Zeiss, Oberkochen, Germany). Stained liver slides were examined at 10× and 20× magnification. Pathological changes in samples from each rat were assessed by examining five to ten selected fields of view. The sections were then photographed.

Histopathological changes were evaluated using semi-quantitative scoring systems. The fibrosis was assessed according to the Ishak score (0–6) and the METAVIR score (F0–F4). The criteria for fibrosis assessment according to the Ishak and METAVIR scoring systems are provided in Tables S3 and S4 of the Supplementary Materials, respectively. Inflammatory changes, necrosis, and bile duct reaction (ductular hyperplasia) were evaluated using a four-point scoring system (0–4), where 0 indicates the absence of pathological changes, 1—minimal changes, 2—moderate changes, 3—marked changes, and 4—severe pathological alterations. Semi-quantitative assessment was performed independently by two pathologists using at least 10 randomly selected fields per rat. The results of histopathological scoring in each experimental group were expressed as median (min–max) values and analyzed using nonparametric statistical methods (Kruskal–Wallis test).

### 2.5. Ultrastructural Analysis of Rat Liver by Transmission Electron Microscopy (TEM)

TEM of rat liver was also used. Liver tissue fragments (2 mm^3^) were first immersed in a 2.5% glutaraldehyde solution prepared in a buffer with a pH 7.4. They were then postfixed in a 1% osmium tetroxide (OsO_4_) solution. The samples were then placed in ethanol solutions of gradually increasing concentrations. After the samples reached 70% ethanol, they were counterstained with a 1% uranyl acetate solution to increase electron density. After dehydration, the material was soaked in the resin mixture, following the embedding procedure. Thin sections were then cut on a UC Enuity ultramicrotome (Leica Microsystems CMS GmbH, Vezlar, Germany), processed with lead citrate according to Reynolds, and examined on a HIMERA EM50X field-emission scanning electron microscope (CIQUTEK, Hefei, Anhui, China). The resulting images were recorded using a digital data acquisition system.

### 2.6. Hydroxyproline Measurement

The protocol began with hydrolysis of liver tissue after which the free hydroxyproline (Hyp) in the resulting hydrolysates is quantified. This quantification was performed via a two-step reaction: chloramine-T reacts with Hyp, then Ehrlich’s reagent is added, producing a chromophore [30]. The absorbance of that chromophore was measured at 550 nm with a spectrophotometer (Ekrokhim LLC, St. Petersburg, Russia). By referencing to calibration curve, the Hyp concentration was expressed in μg per gram of tissue.

### 2.7. Gelatin Zymography

Liver tissue was homogenized in 1 × PBS (pH 7.4) at a ratio of 1:5 (*w*/*v*) and the homogenate was centrifuged at 3000× *g* for 3 min at 4 °C. Total protein in the supernatant was measured using a modified Lowry method [31]. The homogenate was then mixed with 2× Laemmli sample buffer, intentionally excluding β-mercaptoethanol. A total of 50 μg of protein was added to each well of the gel. Samples were then separated by electrophoresis on 10% SDS-PAGE gels copolymerized with 0.2% gelatin [32].

After analysis, the gels were loaded in a renaturation buffer (Invitrogen, Novex, Carlsbad, CA, USA). The gels were allowed to equilibrate and then transferred to the developing buffer (Invitrogen, Carlsbad, CA, USA), where they remained for a full 24 h. The gels were then immersed in 0.25% Coomassie Brilliant Blue R-250 for one hour; excessive dye was washed away with a mixture of isopropanol, acetic acid, and water until the lytic bands became clearly visible against the blue background.

Gelatin degradation zones, which serve as a proxy for matrix metalloproteinase (MMP) activity, were quantified using ImageLab version 6.0.1 (Bio-Rad Laboratories, Hercules, CA, USA). Following electrophoresis, total protein loading was verified using the TGX Stain-Free imaging system according to the manufacturer’s protocol (Bio-Rad, Hercules, CA, USA). The measured intensity of each band was then normalized relative to the signal of the total Stain-Free protein.

### 2.8. Western Blotting

For this analysis, a 1:5 (*w*/*v*) liver homogenate prepared in PBS pH 7.4 with 1 mM PMSF was used. The resulting homogenate was then mixed with 4× Laemmli sample buffer containing 10% β-mercaptoethanol, boiled for 5 min, and centrifuged at 15,000× *g* for 3 min at 4 °C.

To evenly distribute the protein, 10 μg of protein were pipetted into each lane of a 10% Tris-glycine polyacrylamide gel. Separation was performed by electrophoresis. After analysis, the separated proteins were transferred to PVDF membranes (Amersham Pharmacia Biotech, Buckinghamshire, UK) using a Trans-Blot Turbo Transfer System (Bio-Rad, Hercules, CA, USA). Membranes were blocked by incubation with a 5% (*w*/*v*) skim milk solution (SERVA, Heidelberg, Germany) prepared in TBS with 0.05% Tween-20 (PanReac, Barcelona, Spain). They were then incubated with primary antibodies to α-smooth muscle actin (α-SMA) 1:1000 rabbit (ab5694, Abcam, Waltham, MA, USA).

After washing in PBS with 0.05% Triton X-100, the membranes were incubated with secondary antibodies to rabbit IgG conjugated to horseradish peroxidase (1:7500; Imtek, Moscow, Russia). The resulting chemiluminescent signal was detected using a WesternBright ECL kit (Advansta, San Jose, CA, USA). Visualization was performed using a ChemiDocMP imaging system (Bio-Rad, Hercules, CA, USA). To confirm protein loading after electrophoresis, TGX Stain-Free imaging technology (Bio-Rad, Hercules, CA, USA) was used according to the manufacturer’s instructions. Band chemiluminescence intensity was quantified using ImageLab 6.0.1 software (Bio-Rad, Laboratories Inc., Hercules, CA, USA). Protein band intensities were normalized to the TGX protein signal.

### 2.9. Quantitative RT-PCR

We measured mRNA levels of inflammation-related genes (*Il6*, *Tnfa*) using real-time PCR. The 60S ribosomal acidic protein (*Rplp0*) gene was used as a reference for normalization.

Total RNA was isolated from liver tissue using TRIzol reagent (Thermo Fisher Scientific, Carlsbad, CA, USA) according to the manufacturer’s instructions and then processed with DNase I (cat. number EN0525, Thermo Scientific, Waltham, MA, USA, 1000 U/mL). Complementary DNA (cDNA) was obtained using the MMLV RT Kit (Evrogen, Moscow, Russia) according to the manufacturer’s protocol.

Quantitative real-time PCR was performed on a Gentier96E Real-Time PCR System (Xi’an Tianlong Technology Co., Ltd., Xi’an, China) using 5× qPCRmix-HS Master Mix (Evrogen, Moscow, Russia). Primer pairs were designed using the Benchling (San Francisco, CA, USA, https://www.benchling.com/) and Primer-BLAST (NCBI/NLM, Bethesda, MD, USA, https://www.ncbi.nlm.nih.gov/tools/primer-blast/index.cgi, accessed on 28 February 2026) cloud services. All oligonucleotides were subsequently synthesized by DNA-synthesis (Moscow, Russia). Primer sequences are presented in Table S2 of the Supplementary Materials.

### 2.10. Isolation of Liver Mitochondrial Fraction and Total Protein Assay

Mitochondria were isolated from rat liver by differential centrifugation [33]. Fresh liver tissue was homogenized in an ice-cold isolation buffer consisting of 0.25 M sucrose, 20 mM MOPS (pH 7.2), 0.05% fatty acid-free BSA, and 1 mM EGTA. A first centrifugation was at 1500× *g* for 10 min at 4 °C, resulting in the formation of a pellet of nuclei and cellular debris. The supernatant was then transferred to tubes. The mixture was then centrifuged again at a higher speed at 12,000× *g* for 10 min at 4 °C, yielding a mitochondrial-enriched pellet. The mitochondrial pellet was placed in buffered saline solution (150 mM KCl, 20 mM MOPS, pH 7.2) and then diluted to a protein concentration of 40–60 mg/mL. Total protein concentration in this suspension was determined using the Lowry method [31].

### 2.11. Assessment of Lipid Peroxidation and Total Antioxidant Capacity in Liver Mitochondria

The content of TBARS in the mitochondrial fraction of the liver was determined spectrophotometrically at 532 nm [34]. The level of TBARS (nmol/mg total mitochondrial protein) was calculated using a molar extinction coefficient of 156,000 M^−1^ cm^−1^.

Total antioxidant capacity in mitochondria was assessed by measuring the rate of TBARS formation induced by 0.5 mM tert-butyl hydroperoxide (t-BHP) during a 30-min incubation at 37 °C. The reaction was stopped by adding thiobarbituric acid reagent followed by boiling for 1 h. After cooling, absorbance was measured at 532 nm using an Ekros spectrophotometer (Saint Petersburg, Russia). The rate of TBARS formation was expressed as nmol/h × mg of mitochondrial protein.

### 2.12. Determination of Reduced Glutathione and Mixed Protein Disulfides

The levels of GSH in liver tissue were determined spectrophotometrically using Ellman’s reagent at 412 nm [35]. Calibration curves were constructed using GSH standards in the concentration range of 0.1–1.0 mM.

Protein mixed disulfides with glutathione were determined in liver tissue homogenates after protein precipitation with 6% perchloric acid containing 1 mM EDTA and 20 mM NEM. The resulting protein pellet was washed twice with cold acetone and solubilized in 0.1 M Tris-HCl buffer (pH 7.4) containing 6 M urea. Protein disulfides were reduced with 1% NaBH_4_ for 30 min, and the excessive reducing agent was quenched by adding 2 M HCl. The samples were neutralized with 7 M NaOH to reach pH 6–7, and the amount of released glutathione was quantified using an enzymatic recycling assay [36].

### 2.13. Determination of SOD Activity and Glutathione-Related Enzymes

The activity of SOD and glutathione metabolism enzymes was studied in the cytosolic fraction of the liver by spectrophotometric methods. SOD activity was determined by a method based on the inhibition of the superoxide anion radical production rate by SOD, formed in the reaction of quercetin autooxidation in a slightly alkaline medium at 405 nm [37]. One unit of SOD activity was taken as the value corresponding to 50% inhibition of the rate of quercetin autooxidation reaction by SOD.

The activity of glutathione synthetase (GS) was determined using N-acetylcysteine as a substrate in a system of coupled reactions with pyruvate kinase and lactate dehydrogenase [38]. Glutathione reductase (GR) activity was determined through reduction of oxidized glutathione with NADPH [39]. The activity of glutathione peroxidase (GPx) was determined using cumene hydroperoxide as substrate [40]. All measurements were performed at 340 nm on a Zenith 3100 plate reader at 37 °C. To calculate the enzyme activities, the molar extinction coefficient of NAD(P)H (6220 M^−1^cm^−1^) was used.

### 2.14. Statistical and Bioinformatic Data Analysis

The minimum sample size for the experimental groups was determined using power analysis performed in G*Power 3.1.9.7, with a significance level of α = 0.05 and a statistical power of 0.95, resulting in *n* = 5 per group.

Statistical analysis was performed using GraphPad Prism 8 (GraphPad Software Inc., San Diego, CA, USA). The data were tested for normality using the Shapiro-Wilk test and Q-Q plots. Depending on the distribution type, either a parametric one-way analysis of variance (ANOVA) followed by Tukey’s multiple comparison test, or a nonparametric Kruskal–Wallis test followed by Dunn’s multiple comparison test was applied. Results are presented as the mean ± standard error of the mean (SEM). To identify and exclude outliers, we used Grubbs’ and ROUT tests. Differences were considered statistically significant at * *p* < 0.05, ** *p* < 0.01, *** *p* < 0.001.

For principal component analysis (PCA), the data were analyzed using MetaboAnalyst 5.0. Heatmapper (http://heatmapper2.ca/, accessed on 16 February 2026) was used for hierarchical cluster analysis. For the analysis, the mean values of individual parameters were normalized to the mean values in the sham-operated control group, and the common logarithm was performed. The Euclidean distance algorithm and the complete linkage clustering algorithm were selected for heatmap visualization.

## 3. Results

### 3.1. Establishment of the BDL Model and General Experimental Characteristics

In this study, obstructive cholestasis was induced in rats by BDL. Thirty male rats were enrolled in the study at the start of the experiment. Within the first two weeks after surgery, five animals died due to postoperative complications (survival rate of about 80%). The survival dynamics are shown in Figure S1 and are available in the Supplementary Materials.

The remaining 25 rats were divided into groups: 20 animals with BDL and 5 sham-operated control animals (see Section 2.2). Three weeks after BDL (after the development of stable cholestasis and early fibrosis), BDL rats were treated with PA derivatives—PL, PT, and HPA—for three weeks (weeks 3–6). No deaths occurred during the therapy period (weeks 3–6) in any of the treatment groups, and the survival rate during administration of CoA biosynthesis modulators was 100%. At week 6, the animals were euthanized, and blood and liver samples were collected for biochemical and morphological analysis.

Within a few days after surgery, BDL rats developed early characteristic symptoms of acute obstructive cholestasis, including jaundice (yellowing of the skin, ears, and extremities), hyperbilirubinemia and bilirubinuria (see Figure S2, Supplement Files). Serum biochemistry 6 weeks after obstruction and treatment with PL, PT, and HPA confirmed an increase in total bilirubin levels, indicating the development of mechanical jaundice (see Figure S2 in the Supplementary Materials).

It is important to note that rats with BDL did not lose body weight during the 6 weeks of obstruction. Body weight dynamics before surgery, after 3 weeks of obstruction, before and after 3 weeks of treatment with PL, PT, and HPA at week 6 of the experiment are shown in Figure S3 (see Supplementary Materials).

### 3.2. Serum Markers of Cholestatic Liver Injury and Systemic Redox Status in Rats with Obstructive Cholestasis Treated with PA Derivatives

**Figure 2:**
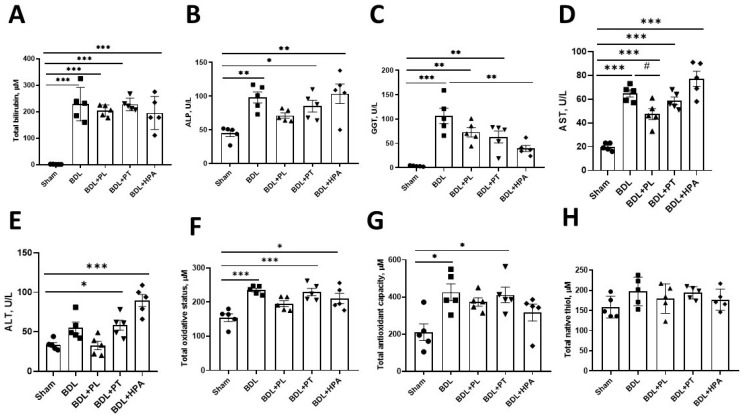
Serum biochemical markers of liver injury and systemic redox status in rats with BDL and administration of PA derivatives. PL, PT, and HPA were administered from week 3 to week 6 after BDL; the study was performed on the day of euthanasia at 6 weeks (**A**) Total bilirubin concentration; (**B**) ALP activity; (**C**) GGT activity; (**D**,**E**) AST and ALT activity, respectively; (**F**–**H**) Serum TOS, TAC, and TSH levels, respectively. Data are presented as mean ± SEM. * *p* < 0.05, ** *p* < 0.01, *** *p* < 0.001 (one-way ANOVA with Tukey’s post hoc test). Each dot in the graph represents the individual value of the measured parameter.

To evaluate liver injury in rats induced by BDL, we analyzed serum biochemical markers of liver function and systemic indicators of the redox balance 6 weeks after obstruction (Figure 2). The main feature of cholestatic liver injury was severe hyperbilirubinemia: the total bilirubin level in serum increased more than 200-fold compared with the control group (Figure 2A). Administration of PA derivatives (PL, PT, and HPA) from weeks 3 to 6 after obstruction did not reduce bilirubin concentration, indicating the pronounced severity of cholestatic injury.

Serum ALP activity was also significantly elevated in all BDL animals (Figure 2B). PL and PT treatment showed only a slight downward trend, while HPA administration had no effect. γ-Glutamyltransferase (GGT) activity increased approximately 100-fold compared with the control group (Figure 2C). Treatment with HPA reduced GGT activity by about twofold, whereas PL and PT showed only minor effect, keeping the values markedly elevated.

To assess the extent of hepatocellular injury under conditions of obstructive cholestasis and following administration of pantothenic acid derivatives, we evaluated serum transaminase activity. Serum AST activity was increased threefold in BDL rats (Figure 2D). Administration of PL to BDL rats resulted in a significant 26% reduction in AST activity, whereas treatment with PT, and particularly HPA, did not lead to a decrease in serum AST levels. A similar pattern was observed for ALT activity in serum, where only PL administration showed a tendency to reduce ALT activity in BDL rats (Figure 2E).

Assessment of systemic redox balance revealed a significant increase in DMPD-reactive products (substrate—DMPD—N,N-dimethyl-p-phenylenediamine), reflecting total oxidant status (TOS) (Figure 2F). Treatment with PA derivatives did not produce statistically significant changes, although PL showed a modest tendency to reduce this parameter. A similar pattern was observed for total antioxidant capacity (TAC) (Figure 2G), which was nearly doubled in all BDL groups, both untreated and treated, likely reflecting compensatory elevation of bilirubin with intrinsic antioxidant properties. The total native thiol content (TSH) in serum remained unchanged across all experimental groups (Figure 2H).

### 3.3. Pathomorphological Changes in the Liver in Obstructive Cholestasis and Its Treatment with PA Derivatives

At the end of the experimental period (6 weeks after BDL), a morphological examination of the rat liver was performed to assess the severity of cholestatic injury and fibrosis. Animals received PA derivatives (PL, PT, or HPA) during the last three weeks following bile duct obstruction.

**Figure 3:**
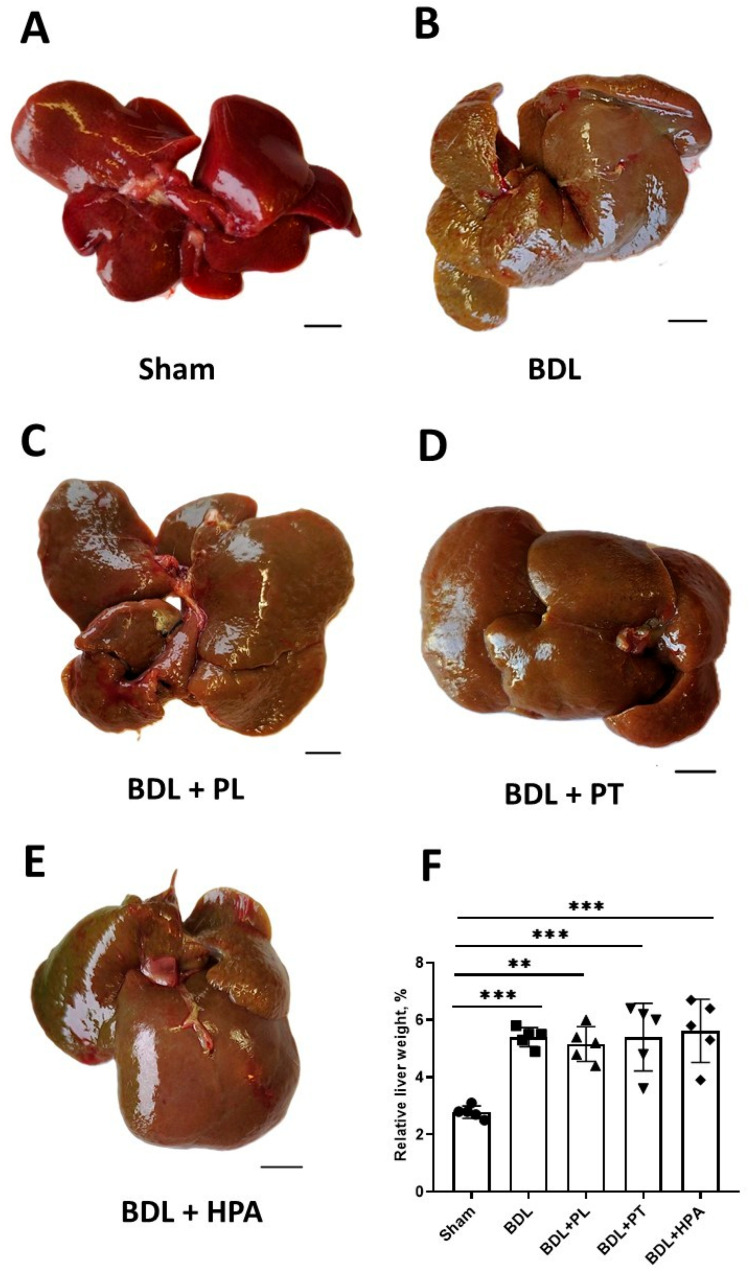
Macroscopic changes in the liver of rats with BDL and administration of PA derivatives. PL, PT, and HPA were administered daily from week 3 to week 6 after BDL; pathomorphological examination was performed 6 weeks after BDL. (**A**–**E**) Representative macroscopic images of the liver. Bar, 1 cm. (**F**) Relative weight of rat liver (in %). ** *p* < 0.01, *** *p* < 0.001 (one-way ANOVA with Dunnett’s post hoc test). Each dot in the diagram (**F**) represents an individual value of the measured parameter.

In sham-operated animals, the liver retained a brownish color with a smooth and glossy capsule, sharp edges, and a soft consistency (Figure 3A). In the BDL group, pronounced macroscopic changes were observed (Figure 3B). The liver became dense and significantly enlarged, with a jaundiced tint and a fine-grained surface. At the site of ligation, the common bile duct was significantly dilated and filled with greenish-yellow bile. All animals in this group exhibited ascitic fluid within the abdominal cavity.

Despite treatment with PA derivatives (PL, PT, and HPA), the pronounced signs of cholestatic liver injury persisted. The drugs did not reduce the degree of hepatomegaly or alleviate the macroscopic manifestations of cholestasis (Figure 3C–E). Quantitative assessment revealed that the relative liver weight in BDL rats was significantly increased on average 1.9-fold compared with controls (Figure 3F). Administration of PA derivatives did not reduce the relative liver weight in rats.

**Figure 4:**
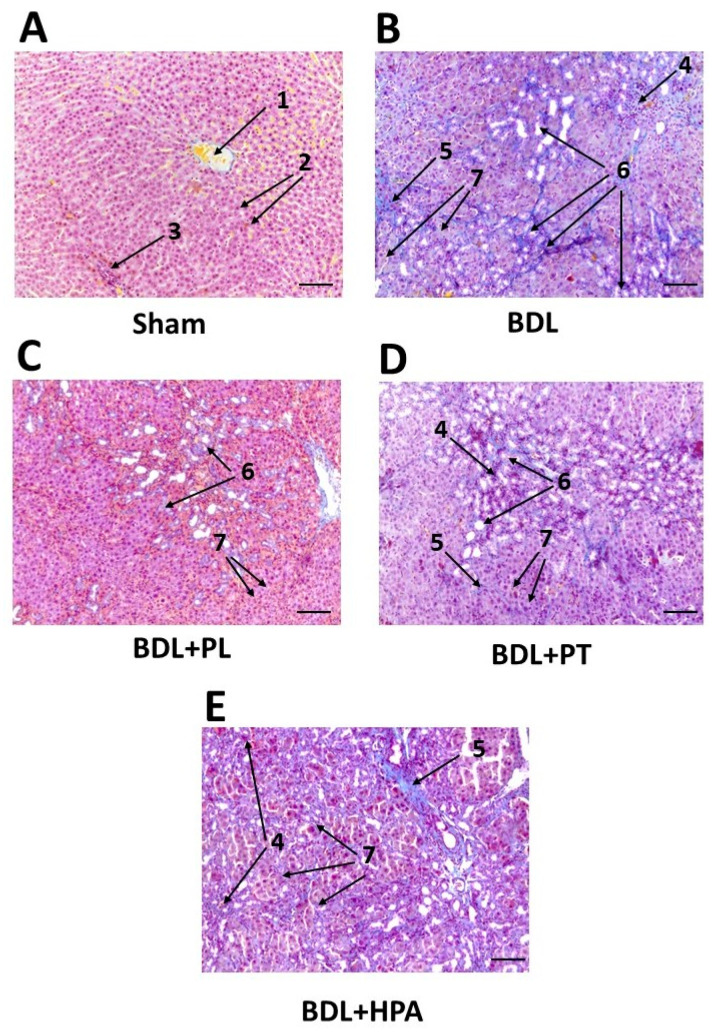
Representative liver sections stained with Mallory’s trichrome. (**A**) Normal liver architecture in sham-operated rats after 6 weeks of laparotomy. (**B**–**E**) Histological alterations after BDL and administration of PA derivatives (from week 3 to week 6 after BDL). The labels on the micrograph correspond to the following structures in the legend: 1—central vein; 2—sinusoidal capillaries; 3—interlobular connective tissue; 4—inflammatory infiltration; 5—connective tissue proliferation; 6—bile duct proliferation; 7—hypertrophic hepatocytes. Arrows indicate the corresponding structures. Bar, 100 µm.

To study pathological changes in liver tissue 6 weeks after BDL and therapy with PA derivatives (from 3 to 6 weeks of obstruction), a histochemical study was performed. Liver tissue was stained using Mallory’s trichrome method to identify connective tissue and assess fibrosis and inflammation (Figure 4).

In sham-operated rats, the liver parenchyma had a characteristic structure consisting of classic hexagonal hepatic lobules (Figure 4A). Hepatocytes formed trabeculae between which sinusoidal capillaries (indicated by the arrowheads) converged toward the center of the hepatic lobule. Central veins were located in the center of the lobules. Portal triads consisting of an interlobular vein, artery, and interlobular bile duct were located at the periphery of the lobules. The boundaries between the lobules were practically indistinguishable due to the small amount of connective tissue.

In rats with BDL, 6 weeks after obstruction, the structure of the liver parenchyma was significantly altered (Figure 4B). There was a strong proliferation of interlobular connective tissue. In the connective tissue interlayers, multiple interlobular bile ducts (marked with arrows) were found with obvious, inflammatory infiltration, and some hepatocytes hypertrophied.

After PL administration (BDL + PL group), the changes in the liver were generally similar to those observed without PL treatment (in the BDL group) (Figure 4C). However, there was an increase in the number of interlobular bile ducts (marked with arrows); the changes were less pronounced than in rats with BDL, and inflammatory infiltration was practically absent, with local isolated hypertrophied hepatocytes.

After treatment with PT (BDL + PT group), the liver morphology also improved, albeit less effectively than after treatment with PL (BDL + PL group) (Figure 4D). However, there was some increase in the size of the interlobular bile ducts (marked with arrows), although the changes were only slightly more pronounced than after PL treatment. There was also pronounced inflammatory infiltration and connective tissue proliferation, and hypertrophied hepatocytes and areas of necrosis were detected.

In contrast, treatment with HPA (BDL + HPA group) did not lead to improvement in BDL-induced injury (Figure 4E). The changes in the liver were similar to those in the BDL group. However, it can be noted that the proliferation of connective tissue and interlobular bile ducts (marked with arrows) was significantly more pronounced, as was the inflammatory infiltration in the duct area.

Thus, treatment of BDL rats with PL and PT was associated with milder histological changes and lower fibrosis markers. Moreover, treatment with PL was more effective than that with PT. HPA aggravated fibrotic changes and worsened liver parenchymal damage.

Additionally, we performed a semi-quantitative assessment of histopathological changes in Mallory-stained liver sections, including fibrosis, inflammation, necrosis, and bile duct reaction, using scoring systems (Table 1).

The degree of fibrosis in all BDL groups, including animals treated with PA derivatives, was comparable and corresponded to stage 4 according to the Ishak scoring system and stage F3 according to the METAVIR scale, indicating portal fibrosis with numerous septa without established cirrhosis. It should be noted that the severity of inflammatory response, necrosis, and bile duct reaction in BDL rats corresponded to a score of 3, reflecting a marked degree of liver injury.

Administration of PL to BDL rats resulted in a moderate reduction in inflammatory response and necrosis, as well as a decrease in bile duct hyperplasia (score 2). In contrast, PT treatment did not substantially affect the degree of fibrosis, although a trend toward reduced bile duct hyperplasia was observed (score 2). HPA did not attenuate pathological changes in BDL rats.

### 3.4. Ultrastructural Alterations of Hepatocytes and Mitochondria in BDL Rats Treated with PA Derivatives

**Figure 5:**
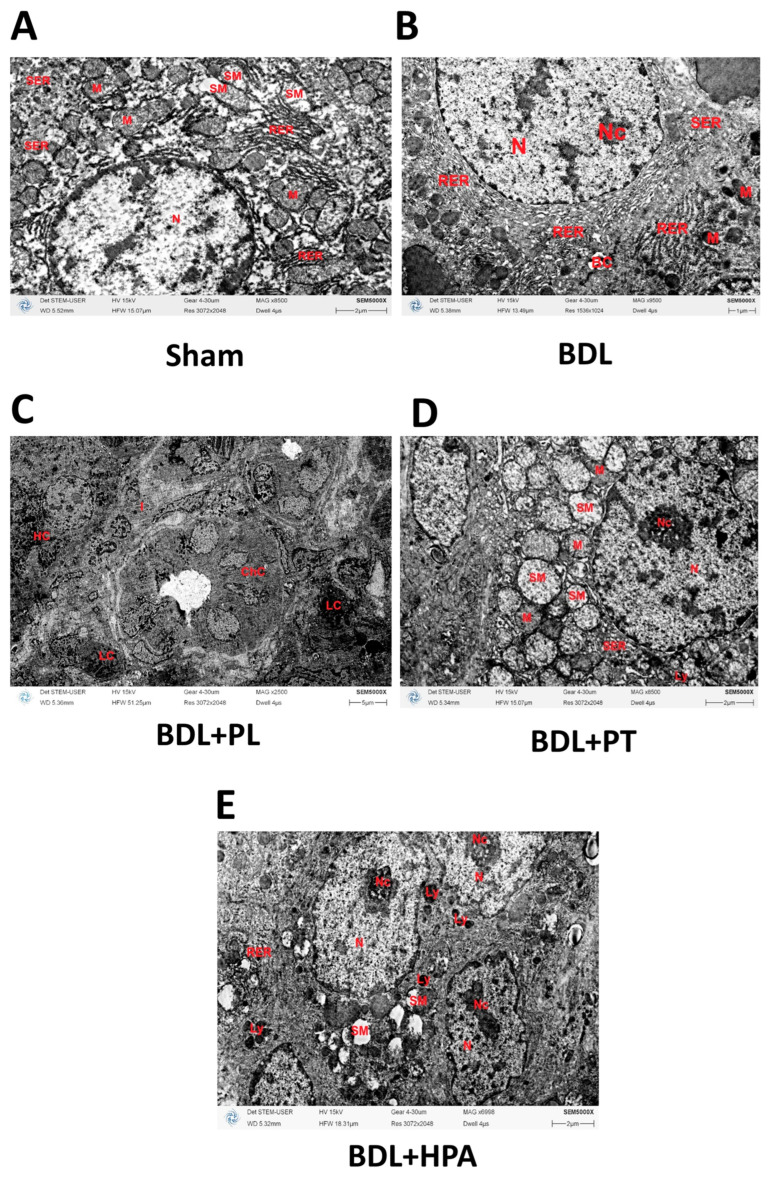
Representative TEM image of hepatocytes. (**A**) Normal hepatocyte architecture in sham-operated rats after 6 weeks of laparotomy. (**B**–**E**) Ultrastructural changes in liver cells after BDL and administration of PA derivatives (PL, PT and HPA treatment from week 3 to week 6 after BDL). Abbreviations: BC—bile canaliculus; ChC—cholangiocyte; I—Ito cell (hepatic stellate cell); HC—hepatocyte; LC—leukocyte; Ly—lysosome; M—mitochondria; N—nucleus; Nc—nucleolus; RER—rough endoplasmic reticulum; SER—smooth endoplasmic reticulum; SM—swollen mitochondria. Bar, 2 μm (**A**); 1 μm (**B**); 5 μm (**C**); 2 μm (**D**,**E**).

To further evaluate subcellular alterations associated with cholestatic liver injury, an ultrastructural analysis of hepatocytes was performed using transmission electron microscopy (TEM). In sham-operated rats, the ultrastructural organization of hepatocytes corresponded to normal liver architecture. Hepatocytes were polygonal, with centrally located, round euchromatic nuclei containing distinct nucleoli. Mitochondria were large and rounded, with short lamellar cristae and an electron-lucent, fine-granular matrix, closely associated with rough endoplasmic reticulum (RER). Both RER and smooth endoplasmic reticulum (SER) were moderately developed and exhibited no cisternal dilatation. Occasional mild mitochondrial swelling and a few lipid droplets were observed. The Golgi apparatus was poorly developed, whereas numerous glycogen granules were present in the cytoplasm (Figure 5A).

In the BDL group, pronounced ultrastructural abnormalities were observed. There was a marked increase in the number of bile ducts, and their lumina contained dense bile material and pigment deposits. Cholangiocytes nuclei contained large nucleoli, indicating active protein synthesis typical for proliferating cells. Hepatocytes exhibited condensed mitochondria with a reduced profile, increased matrix electron density, swollen cristae, and electron-dense inclusions within the matrix. Both SER and RER showed pronounced hyperplasia, with expanded cisternae and a decreased number of ribosomes and polysomes. Cytoplasmic accumulation of bile pigment droplets was observed, while lipid inclusions were significantly reduced as compared with the control. The nuclear envelope was wrinkled, and the nuclear shape was irregular; isolated apoptotic nuclei displayed chromatin condensation beneath the nuclear envelope and nuclear deformation or fragmentation (Figure 5B).

In the BDL + PL group, the number of bile ducts remained elevated. The lumina of bile canaliculi were dilated and filled with bile and bile pigment, while the microvilli were flattened or reduced. Similar changes were observed in the lumina of several bile ducts. Scattered infiltration by polymorphonuclear leukocytes was found beneath the basal lamina of the bile ducts (Figure 5C). The nuclei of hepatocytes retained normal morphology and contained large nucleoli. The morphology of the endoplasmic reticulum resembled that in the BDL group; however, a greater number of attached ribosomes and polysomes were observed. Bile pigment inclusions in the cytoplasm were rare, and the Golgi apparatus remained poorly developed. Mitochondria had shortened, partially swollen cristae, and the electron density of the matrix varied within individual cells from high to moderate.

In the BDL + PT group, hepatocytes contained large mitochondria with a fine-granular, electron-lucent matrix and few cristae. Local swelling of individual mitochondria or their clusters was observed, with matrix clearing and partial cristae destruction. Nuclei were round and contained from two to three prominent nucleoli. The ultrastructure of the endoplasmic reticulum was similar to that in the control group (Figure 5D). No signs of bile duct hyperplasia were detected.

In contrast, hepatocytes of the BDL + HPA group showed pronounced degenerative changes. Mitochondrial swelling was evident, accompanied by matrix clearing and partial destruction of cristae. Nuclear deformation, marginal chromatin condensation, and the presence of large nucleoli were observed. Both RER and SER were moderately developed, and numerous lysosomes were seen in the cytoplasm, indicating activation of degradative processes (Figure 5E).

### 3.5. Changes in Molecular Markers of Fibrogenesis in the Liver After Treatment with Modulators of PA Derivatives

**Figure 6:**
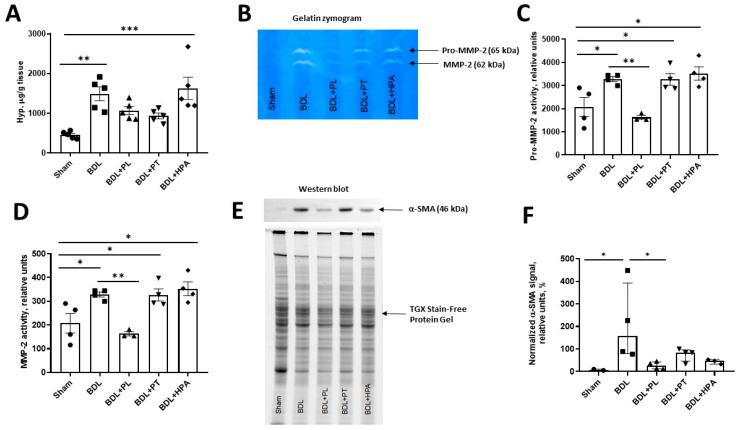
Molecular markers of hepatic fibrosis in the liver of rats with chronic obstructive cholestasis (6-week BDL) and administration of PA derivatives (PL, PT, and HPA from week 3 to week 6 after BDL). (**A**) Hepatic Hyp content; (**B**) Representative gelatin zymogram showing Pro-MMP-2 (65 kDa) and MMP-2 (62 kDa) activity in liver samples; (**C**,**D**) Densitometry quantification of activity of Pro-MMP-2 (65 kDa) and mature MMP-2 (62 kDa) in liver tissue; (**E**) Representative Western blot image for α-SMA (46 kDa) and loading control using a TGX Stain-Free gel in liver lysate samples; (**F**) Normalized densitometry analysis of α-SMA-specific bands. * *p* < 0.05, ** *p* < 0.01, *** *p* < 0.001 (one-way ANOVA with Tukey’s post hoc test). Each dot in the graph represents the individual value of the measured parameter.

The morphological changes, which we observed in the liver tissue of rats with BDL, were associated with the development of fibrosis. To elucidate the molecular mechanisms and evaluate fibrosis, we examined additional indicators of fibrogenesis. Specifically, we analyzed Hyp levels, matrix metalloproteinase (MMP) activity, and α-smooth muscle actin (α-SMA) expression in liver tissue samples (Figure 6).

In rats exposed to BDL only, the Hyp content in the liver increased 3-fold compared with the control group. Treatment with PL and PT reduced the level of Hyp, while HPA had no noticeable effect, and the level of Hyp remained significantly elevated (Figure 6A).

A similar pattern was observed in the analysis of MMP activity, assessed by gelatin zymography (Figure 6B). In the BDL group, the activity of both pro-MMP-2 and mature MMP-2 was increased 1.6-fold compared with the sham-operated group. Treatment with PL, unlike PT and HPA, shifted MMP activity to values close to those in the control group (Figure 6C,D).

In addition, Western blotting revealed a significant increase in α-SMA expression in the livers of rats with BDL, suggesting activation of hepatic stellate cells and the onset of fibrogenesis. PL treatment significantly reduced α-SMA expression, whereas PT and HPA administration did not result in significant improvements (Figure 6E,F).

Thus, PL showed a more notable association with reduced fibrotic markers compared with PT, although fibrosis remained clearly present in all BDL groups, and HPA was even associated with worsening fibrosis.

### 3.6. Assessment of Inflammation, Oxidative Stress, and Antioxidant System Activity in the Liver of BDL Rats Treated with PA Derivatives

**Figure 7:**
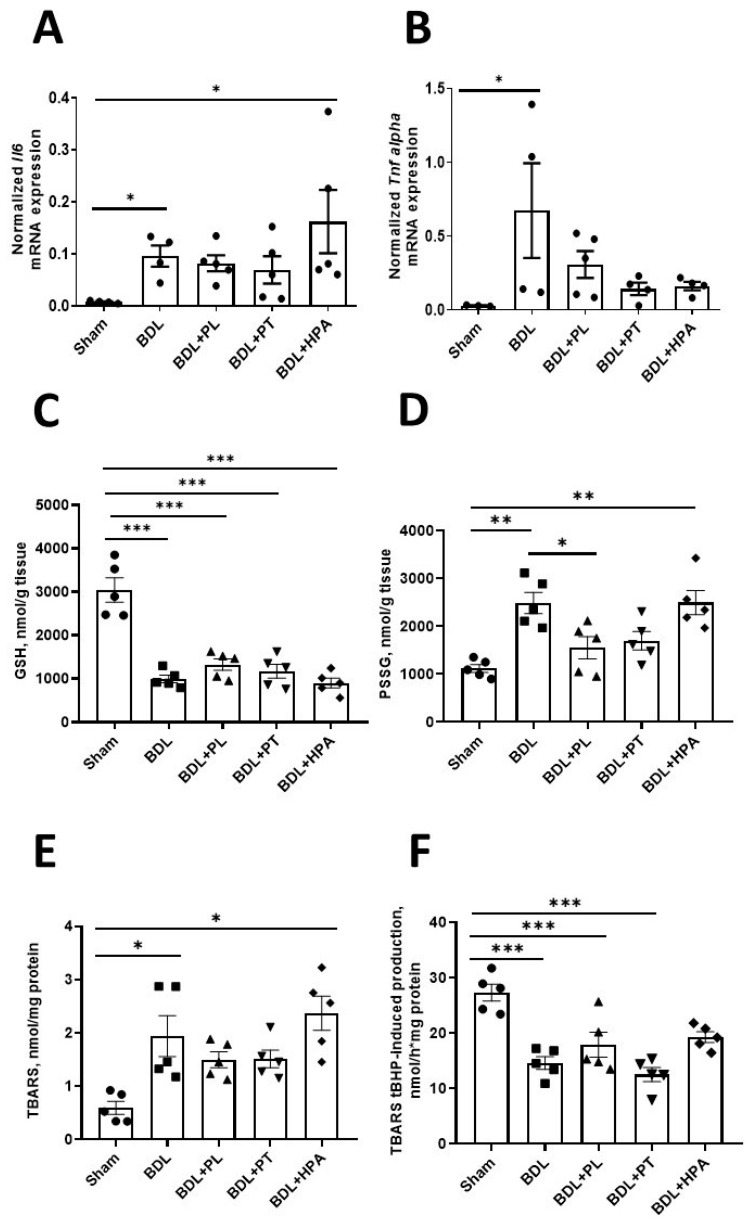
Response of inflammatory and oxidative stress markers in the liver mitochondria of rats with chronic obstructive cholestasis (6-week BDL) and administration of PA derivatives (PL, PT, and HPA from week 3 to week 6 after BDL). (**A**,**B**) Relative expression of mRNA encoding IL-6 and TNF-α in liver tissue; (**C**) GSH content in liver tissue; (**D**) PSSG levels in liver tissue; (**E**) Basal level of TBARS in liver mitochondria; (**F**) tBHP-induced production of TBARS in liver mitochondria. * *p* < 0.05, ** *p* < 0.01, *** *p* < 0.001 (one-way ANOVA with Tukey’s post hoc test). Each dot in the graph represents the individual value of the measured parameter.

Since we confirmed signs of liver cell damage and fibrosis development using morphological methods, we additionally studied markers of inflammation and oxidative stress to substantiate the mechanisms of the hepatoprotective action of PA derivatives (Figure 7).

We found a significant increase in the expression of mRNA coding for the pro-inflammatory cytokines IL-6 and TNF-α in the livers of rats exposed to BDL (Figure 7A,B). Treatment with PL, PT (but not HPA) showed a tendency to decrease TNF-α expression; however, it did not reach control values. In contrast, HPA did not reduce inflammation in the liver of rats with BDL, as evidenced by the increased level of mRNA expression of IL-6.

Significant changes were also observed in the glutathione system, which is a key component of the liver’s antioxidant defense. In all BDL groups, both untreated and treated with PA derivatives, the hepatic content of reduced glutathione (GSH) was decreased threefold compared with controls (Figure 7C). Administration of PL, PT, or HPA did not normalize the GSH level. Concomitant with GSH depletion, there was a marked increase in mixed protein conjugates with glutathione (PSSG), a marker of protein S-glutathionylation (Figure 7D). In BDL rats, hepatic PSSG levels were more than doubled. PL, and to a lesser extent PT, significantly reduced PSSG content toward control values, whereas HPA had no noticeable effect. Thus, under conditions of chronic destructive liver alterations accompanied by pronounced oxidative stress and severe depletion of GSH levels, it appears unrealistic to achieve complete restoration of GSH solely through the potential antioxidant effects of some PA derivatives. Importantly, under such conditions, the level of PSSG appeared to be among the most sensitive indicators reflecting the modulatory action of PA derivatives.

We further examined oxidative stress manifestations in liver mitochondria by measuring basal and tert-butyl hydroperoxide (tBHP)-induced thiobarbituric acid reactive substances (TBARS) formation. The basal level of TBARS in hepatic mitochondria was increased fivefold in BDL rats, including those treated with HPA (Figure 7E). Treatment with PL or PT resulted in a moderate decrease in basal TBARS, although the values remained considerably higher than in controls.

Assessment of the mitochondrial antioxidant potential, based on inhibition of tBHP-induced TBARS formation in vitro, revealed a significant reduction in this parameter in all BDL groups, regardless of treatment (Figure 7F). These findings suggest an altered susceptibility of hepatic mitochondria to oxidative damage ex vivo, likely due to adaptive activation of antioxidant defense systems under conditions of pronounced oxidative stress in vivo.

**Figure 8:**
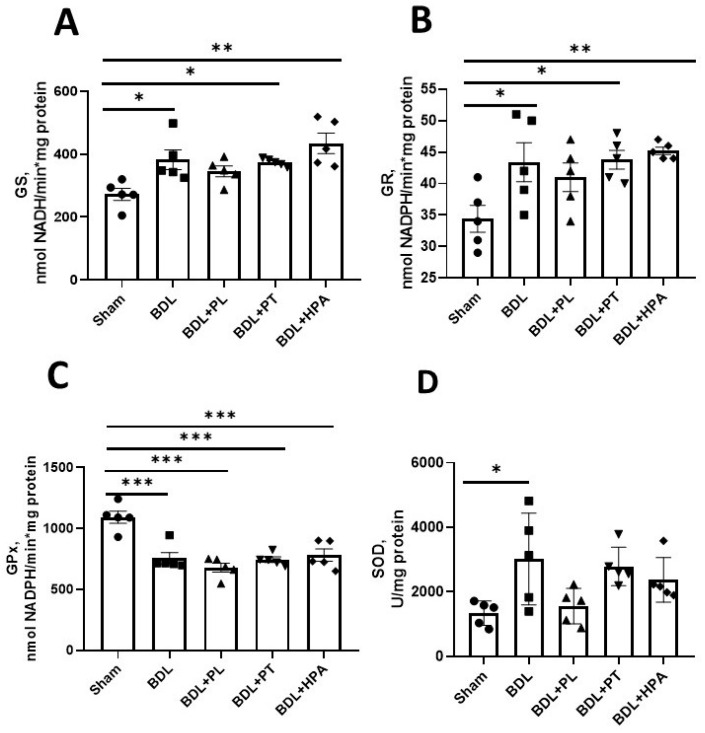
Activity of glutathione metabolism enzymes and SOD in the cytosolic fraction of the liver of rats with chronic obstructive cholestasis (bile duct ligation for 6 weeks) and administration of PA derivatives (PL, PT, and HPA from the 3rd to the 6th week after bile duct ligation). (**A**) Glutathione synthetase activity; (**B**) Glutathione reductase activity; (**C**) Glutathione peroxidase activity; (**D**) SOD activity. * *p* < 0.05, ** *p* < 0.01, *** *p* < 0.001 (one-way analysis of variance with Tukey’s post hoc test). Each point on the graph represents an individual value of the measured parameter.

Profound depletion of the hepatic GSH pool in rats with obstructive cholestasis was accompanied by a 1.5-fold increase in glutathione synthetase (GS) activity in the cytosolic liver fraction (Figure 8A). In addition, a moderate elevation of cytosolic glutathione reductase (GR) activity was observed in all BDL rats (Figure 8B). However, administration of PA derivatives to BDL rats did not significantly alter the activity of enzymes involved in GSH biosynthesis.

Cytosolic glutathione peroxidase (GPx) activity was reduced by approximately 30% in all BDL groups compared with the Sham group, whereas cytosolic superoxide dismutase (SOD) activity was increased by about 40% (Figure 8C,D). Treatment with PA derivatives did not markedly affect GPx or SOD activities. Only PL administration in BDL rats showed a pronounced trend toward a reduction in SOD activity.

Overall, under conditions of chronic oxidative stress and inflammation induced by BDL, PA derivatives exhibited limited therapeutic efficacy. Among the redox-related parameters examined, protein-glutathione mixed disulfides appeared to be the most sensitive to treatment. PL (and to a lesser extent PT) most effectively prevented the formation of protein-glutathione conjugates, which may indicate their protective effects. In contrast, HPA did not improve and, in some cases, exacerbated markers of inflammation and oxidative stress in the liver of rats with obstructive cholestasis.

### 3.7. Principal Component Analysis and Integrated Visualization of Hepatic Biochemical and Molecular Responses in BDL Rats Treated with PA Derivatives

To integrate the biochemical, morphological, and molecular data and to identify interrelationships among the key pathogenetic mechanisms underlying cholestatic liver injury, we performed multivariate statistical analysis. Specifically, principal component analysis (PCA) and hierarchical clustering were applied to visualize associations among serum markers of liver injury, oxidative stress, inflammation, and fibrogenesis, as well as to differentiate the experimental groups treated with PA derivatives (PL, PT, and HPA). In addition, a heatmap was generated to illustrate the biochemical profiles associated with the administration of pantothenic acid derivatives in the setting of obstructive cholestasis.

**Figure 9:**
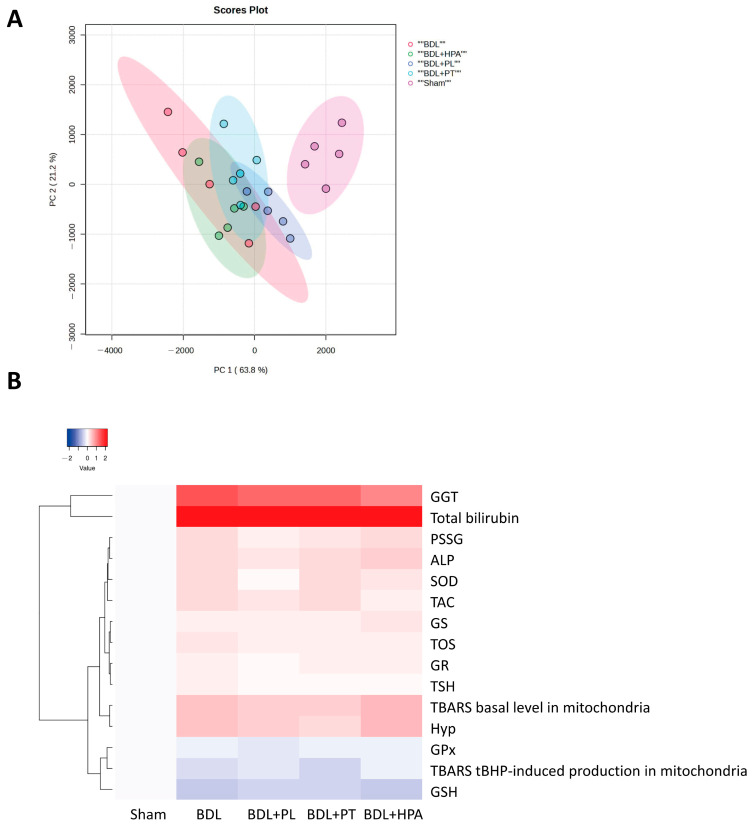
Effect of treatment with PA derivatives (PL, PT, and HPA) in rats with 6-week BDL on systemic changes in biochemical parameters of liver injury, fibrosis, oxidative stress, and glutathione system. BDL treatment with PA derivatives was performed from 3 to 6 weeks after obstruction. (**A**) Visualization of principal component analysis to evaluate biochemical changes in serum and liver tissue after treatment with BDL with PA derivatives. (**B**) Cluster analysis and heat map to evaluate systemic biochemical changes in blood and liver after treatment with BDL with PA derivatives. Abbreviations: GGT—γ-Glutamyltransferase; PSSG—Mixed protein disulfides with glutathione; ALP—Alkaline phosphatase; SOD—Superoxide dismutase; TAC—Total antioxidant capacity; GS—Glutathione synthetase; TOS—Total oxidant status; GR—Glutathione reductase; TSH—Total native thiol; TBARS—Thiobarbituric acid reactive substances; Hyp—Hydroxyproline; GPx—Glutathione peroxidase; GSH—Reduced glutathione.

Multivariate analysis using PCA revealed marked differences in biochemical parameters between rats subjected to 6 weeks of bile duct ligation (BDL) and sham-operated animals. Administration of PL and PT for 3–6 weeks after bile duct obstruction moderately ameliorated these alterations, whereas HPA aggravated liver injury (Figure 9A). During BDL progression, PL- and PT-treated groups demonstrated clear clustering and separation from the untreated BDL group, while the HPA-treated BDL group substantially overlapped with the BDL group.

Furthermore, hierarchical clustering based on hepatic biochemical parameters reflected pronounced differences in the degree of fibrosis and oxidative stress. Notably, PL and PT clustered separately from untreated BDL rats and were characterized by relatively lower fibrosis and oxidative stress markers; however, given the sample size, these associations should be interpreted as descriptive rather than predictive (Figure 9B) and should be considered with caution. Particular attention should be paid to the first cluster, which includes parameters reflecting the severity of cholestatic liver injury in BDL rats, specifically total bilirubin levels and GGT activity. This cluster represents the high degree of liver damage of the chronic obstructive cholestasis model.

In contrast, the cluster comprising fibrosis and oxidative stress markers (hepatic hydroxyproline content and basal mitochondrial TBARS levels), as well as the cluster integrating antioxidant status parameters (GSH levels, GPx activity, and tBHP-induced TBARS formation in hepatic mitochondria), appeared to be the most responsive to PA derivatives. Thus, treatment with PA derivatives moderately reduced hepatocyte damage in obstructive cholestasis, mainly by modulating parameters related to redox balance.

Cluster analysis thus enabled the identification of sensitive indicators reflecting potential biochemical pathways associated with the protective effects of PA derivatives. However, these associations are not interpreted here in a strictly mechanistic context. The precise molecular mechanisms underlying the protective effects of PA derivatives in cholestasis require further investigation.

## 4. Discussion

Our results suggest that BDL-induced chronic obstructive cholestasis is associated with a complex spectrum of metabolic, redox, and fibrotic disturbances in rat liver, consistent with previously described experimental models of cholestatic injury [41]. Since the administration of PA derivatives was initiated at the third week after BDL, at a stage characterized by pronounced fibrotic changes, we expected that the treatment would not lead to a reduction in markers of mechanical biliary obstruction. Rather, it was expected to modulate secondary pathogenetic mechanisms associated with cholestasis progression and oxidative stress. Our hypothesis was supported by biochemical changes in rat blood. Serum markers of hepatocellular damage and cholestasis (total bilirubin, ALP, and GGT) remained markedly elevated in BDL rats. On the other hand, we showed that liver morphology and other pathological features may be alleviated after pharmacological treatments. PL and, to a lesser extent, PT improved liver morphology, attenuated fibrosis, and prevented mitochondrial damage, suggesting partial restoration of cellular architecture independently of bile flow recovery. Notably, PL and PT, but not HPA, significantly reduced bile duct hyperplasia, mitigated mitochondrial and ER structural abnormalities, and thus appear to target downstream consequences of cholestasis progression rather than its purely mechanical origin.

Ultrastructural examination of hepatocytes from rats with chronic cholestasis revealed severe degenerative alterations associated with disruption of mitochondrial membranes and decreased electron density of the mitochondrial matrix. Such morphological abnormalities are known to impair mitochondrial functional activity and may synergize with the cytotoxic effects of accumulated BAs [42]. BAs activate inflammatory signaling cascades involving NF-κB and TGF-β, leading to HSC activation [43]. Additionally, due to their detergent properties, BAs disrupt mitochondrial membrane integrity and exert uncoupling effects. These molecular events promote hepatocellular membrane damage and ECM remodeling in cholestatic liver tissue [18]. PL and PT, but not HPA, significantly improved mitochondrial membrane integrity, restored matrix electron density, and reduced mitochondrial swelling in hepatocytes from BDL rats. The preservation of mitochondrial ultrastructure in PL- and PT-treated rats was accompanied by reduction in TBARS levels, suggesting an association between mitochondrial integrity and decreased lipid peroxidation, as previously demonstrated in various in vitro and in vivo models of oxidative stress [14,17,44].

Mitochondrial dysfunction is widely recognized as a central mechanism in cholestatic liver injury. The accumulation of hydrophobic bile acids disrupts respiratory chain activity, enhances ROS generation, and activates signaling pathways leading to apoptosis or necrosis of hepatocytes [45]. Our findings corroborate this mechanism through both ultrastructural evidence and molecular markers of oxidative mitochondrial damage. Specifically, BDL rats exhibited a threefold increase in TBARS levels and a marked decline in total mitochondrial antioxidant capacity following ex vivo exposure to tBHP, indicating pronounced oxidative stress [46]. PL and PT tended to reduce lipid peroxidation, whereas HPA failed to prevent mitochondrial oxidative injury, in agreement with ultrastructural observations.

Chronic obstructive cholestasis was also associated with profound alterations in GSH homeostasis. A dramatic depletion of GSH in BDL rats was accompanied by significant upregulation of cytosolic GSH biosynthetic enzymes, suggesting compensatory activation. Administration of PA derivatives did not restore GSH levels or normalize enzyme activities, despite previous evidence that PA and PL can stimulate glutathione biosynthesis in lymphoblastoid cells [16,47]. These discrepancies likely reflect cell-type-specific regulatory mechanisms and differences in redox adaptation under severe oxidative stress. Importantly, PL and PT—but not HPA—reduced hepatic levels of mixed protein disulfides (PSSG), which may reflect partial modulation of GSH homeostasis during chronic oxidative stress in BDL liver. PL is known to exhibit moderate antioxidant activity, which may contribute to partial stabilization of redox balance in the liver and protection of proteins from oxidative damage. This is associated with stabilization of lipid peroxidation products and modulation of protein modifications via S-glutathionylation. It is possible that PL, acting as a scavenger of reactive oxygen species, reduces their accumulation and thereby decreases the level of S-glutathionylated proteins in the liver of BDL rats. We have previously demonstrated that PL exerts protective effects through modulation of thiol-disulfide redox balance, leading to decreased protein S-glutathionylation in models of rhabdomyolysis, aluminum toxicity, and rotenone-induced neurotoxicity [14,48]. It is plausible that similar mechanisms underlie the protective effects of PL in cholestatic liver, although further mechanistic studies are warranted.

As expected, BDL induced classical periportal fibrosis characterized by proliferation of interlobular connective tissue, activation of HSCs, and ECM remodeling. These findings were supported by increased hepatic Hyp content, elevated α-SMA expression, and altered gelatinase activity assessed by gelatin zymography. Comparable fibrotic profiles have been described in methodological and experimental studies of BDL-induced liver fibrosis [49,50].

Among the tested compounds, PL exhibited pronounced antifibrotic activity, reflected by reduced Hyp content, normalization of MMP-2 and pro-MMP-2 activity, and decreased α-SMA expression. These effects may be attributed to the antioxidant properties of PL in BDL conditions, leading to suppression of HSC activation and attenuation of ECM remodeling. PL also possesses anti-inflammatory properties associated with reduced expression of proinflammatory cytokines, including TNF-α and IL-6, consistent with our findings. In addition, modulation of NF-κB signaling by PL may contribute to attenuation of inflammation and prevention of hepatocyte apoptosis [11,12,51].

Overall, the observed alterations in serum biochemical markers and multilevel morphological changes (macro-, micro-, and ultrastructural) in BDL rats are consistent with advanced-stage liver fibrosis. Under conditions of chronic oxidative stress and inflammation, PL and PT—but not HPA—exerted moderate hepatoprotective effects. These findings are supported by PCA of integrated biochemical parameters, where PL and PT demonstrated partial protective clustering, whereas HPA showed no protective effect and was associated with aggravation of hepatic injury in BDL rats.

The differential antifibrotic effects of PL, PT, and HPA provide insight into distinct mechanisms of action, potentially related to their biotransformation and intracellular metabolism in hepatocytes. PL, which is efficiently converted to PA, participates in the CoA biosynthetic pathway and exhibits pronounced protective effects. However, these effects cannot be directly attributed to CoA-related mechanisms. PT requires extracellular hydrolysis by vanin-1, which may limit its bioavailability under conditions of chronic oxidative stress and inflammation, resulting in weaker efficacy. In contrast, HPA lacks protective properties and is consistent with its antagonistic and inhibitory effects on cellular energetics and the CoA system in hepatocytes [24]. Our findings suggest that free thiol availability may represent a critical determinant of hepatocyte resistance to oxidative stress and fibrosis development during obstructive cholestasis. The CoA system, as a component of thiol-based antioxidant defense, may represent a potential link between PA and its derivatives with redox regulation and protection of mitochondria from oxidative stress during liver fibrosis. However, clarification of this association requires further investigation.

Our results have several limitations that should be considered when interpreting the findings. First, the study was performed in 7–9-month-old male rats, based on the assumption that cholestatic liver diseases caused by biliary obstruction are more prevalent in adult and elderly patients, which may support the translational relevance of the BDL model to clinical settings [52]. Although cholestatic disorders are more frequently observed in women, the BDL model in this study was established in male rats to minimize potential variability associated with sex hormone–dependent redox and metabolic changes. The relatively small sample size in the experimental groups (*n* = 5) may represent a limitation when assessing the efficacy of treatment with pantothenic acid derivatives. However, the minimum sample size was determined a priori using power analysis (G*Power 3.1.9.7) and is consistent with established ethical standards for animal research. Second, obstructive cholestasis in clinical practice is more often chronic rather than acute [53]. Accordingly, in the model used in this study, drug administration was initiated three weeks after bile duct ligation, i.e., at a stage characterized by pronounced inflammation and progressive liver fibrosis. By the sixth week after obstruction, rats developed advanced-stage liver fibrosis accompanied by inflammation, necrosis, persistent oxidative stress, and marked suppression of the glutathione system. Third, under conditions of chronic and severe hepatocellular injury induced by BDL, the therapeutic effects of PA derivatives could not influence the mechanical component of the obstruction and were likely limited to modulation of secondary pathogenic mechanisms. In this context, the moderate changes observed in glutathione system parameters, associated with a reduction in mixed protein disulfide levels, should be interpreted as adaptive redox responses under conditions of severe cholestatic injury.

It is also important to consider the dosing strategy of the investigated compounds. PL was administered at a dose of 500 mg/kg, consistent with previously published studies, whereas PT and HPA were administered at equimolar doses calculated on the basis of pantothenic acid (mmol/kg). The weaker effect of PT compared with PL may be related to its metabolic biotransformation, which includes reduction to pantetheine, hydrolysis to pantothenate mediated by Vanin enzymes, and subsequent phosphorylation to 4′-phosphopantetheine. Finally, it cannot be excluded that earlier initiation of therapy with PA derivatives might have resulted in more pronounced protective effects, which warrants further investigation.

It should be noted that treatment with pantothenic acid derivatives was primarily aimed at correcting metabolic processes underlying redox-dependent and antifibrotic pathways in the liver and was not intended to address the primary mechanical cause of cholestasis in the BDL model.

## 5. Conclusions

In a model of advanced chronic obstructive cholestasis in rats, characterized by established fibrosis, inflammation, oxidative stress, and pronounced impairment of the GSH system, PL and PT did not reduce the severity of cholestasis but attenuated the progression of fibrosis, whereas HPA promoted its progression. PL and PT were associated with partial attenuation of bile duct hyperplasia, inflammatory changes and ultrastructural alterations in mitochondria, while not affecting the severity of cholestasis. In contrast, HPA exacerbated liver damage with obstructive cholestasis. The protective effects of PL and PT may be attributed to their antioxidant properties, leading to moderate reduction of mitochondrial lipid peroxidation and modulation of the GSH system through mobilization of its pool of mixed protein disulfides. Acting as a functional antagonist of PA, HPA largely counteracted the hepatoprotective effects of PL and PT, thereby contributing to aggravation of liver injury severity in cholestatic rats.

## Figures and Tables

**Table 1:** Comparative semi-quantitative assessment of pathological changes in the liver of rats with BDL and treatment with pantothenic acid derivatives (PL, PT, and HPA). Values are presented as median (min–max).

Groups	Ishak Fibrosis Score ^1^ (0–6)	METAVIR Fibrosis Score ^1^ (F0–F4)	Inflammation Score ^2^ (0–4)	Necrosis Score ^2^ (0–4)	Bile Duct Reaction Score ^2^ (0–4)
Sham (*n* = 5)	0 (0–0)	F0 (0–0)	0 (0–0)	0 (0–0)	0 (0–0)
BDL (*n* = 5)	4 (4–5)	F3 (3–3)	3 (3–3)	3 (2–3)	3 (3–3)
BDL + PL (*n* = 5)	4 (3–4)	F3 (3–3)	2 (2–3)	2 (2–2)	2 (2–2)
BDL + PT (*n* = 5)	4 (4–4)	F3 (3–3)	3 (3–3)	3 (2–3)	2 (2–3)
BDL + HPA (*n* = 5)	4 (4–5)	F3 (3–3)	3 (2–3)	3 (3–3)	3 (3–3)

^1^ The criteria for fibrosis staging according to the Ishak and METAVIR scoring systems are provided in Supplementary Tables S3 and S4, respectively. ^2^ Inflammation, necrosis, and bile duct reaction were assessed using a semi-quantitative scale (0–4), as described in Section 2.4 (Materials and Methods).

## Data Availability

The original contributions presented in this study are included in the article/Supplementary Materials. Further inquiries can be directed to the corresponding authors.

## Supplementary Materials

The following supporting information can be downloaded at: https://www.mdpi.com/article/10.3390/pathophysiology33020032/s1, Figure S1: Survival dynamics of rats after common bile duct ligation (BDL) during the first three weeks after obstruction; Survival in sham-operated rats (*n* = 5) was 100%. In rats with BDL (*n* = 25), survival was 80% (week 3 of the experiment)—5 rats out of 25 died. Two rats died due to non-specific postoperative complications, and three rats died due to progressive acute cholestasis and sepsis; Figure S2: Symptoms of obstructive cholestasis in rats. (A) Jaundice. (B) Bilirubinuria (qualitatively confirmed by rapid urine test, Siemens Multistix, 10 SG, New York, NY, USA). (C) Serum icterus. (D) Autopsy of a rat with BDL 6 weeks after obstruction. Note: The results of the biochemical analysis of rat blood serum are presented in Figure 2 of the manuscript (Section 3.2, “Results”); Figure S3: Dynamics of changes in the weight of sham-operated rats and rats with common bile duct ligation (BDL) before and after treatment with PA derivatives—panthenol (PL), pantethine (PT), hopantenic acid (HPA); Figure S4: Gelatin zymography. Polyacrylamide gel with gelatin as a substrate. To assess MMP activity, the gel was stained with Coomassie R-250. Collagenase II was used as a positive control. The fragment presented in the manuscript was highlighted with a dotted frame; Figure S5: Evaluation of alpha-smooth muscle actin (α-SMA) expression by Western blotting. The image shows a developed PVDF membrane. The dotted frame highlights a fragment for representative illustration. The molecular weights of the marker proteins are indicated by arrows on the right; Figure S6: TGX Stain-Free Protein Visualization Gel; Table S1: Number of rats in each experimental group for specific protocols and methods; Table S2: Primer sequences used to estimate gene expression; Table S3: Criteria for the Ishak fibrosis scoring system [54]; Table S4: Criteria for the METAVIR fibrosis staging system [55].

## Abbreviations

The following abbreviations are used in this manuscript:

ABTS	2,2′-Azino-bis(3-ethylbenzothiazoline-6-sulfonic acid)
ALP	Alkaline phosphatase
BA	Bile acid
BDL	Bile duct ligation
CoA	Coenzyme A
DMPD	N,N-dimethyl-p-phenylenediamine
ECM	Extracellular matrix
GABA	γ-Aminobutyric acid
GGT	γ-Glutamyltransferase
GPx	Glutathione peroxidase
GR	Glutathione reductase
GS	Glutathione synthetase
GSH	Reduced glutathione
HPA	Hopantenic acid
Hyp	Hydroxyproline
IL-6	Interleukin-6
MMP	Matrix metalloproteinase
NF-κB	Nuclear factor kappa B
PANK	Pantothenate kinase
PCA	Principal component analysis
PL	Panthenol
PT	Pantethine
PSSG	Mixed protein disulfides with glutathione
ROS	Reactive oxygen species
α-SMA	Alpha-smooth muscle actin
SOD	Superoxide dismutase
TAC	Total antioxidant capacity
TBARS	Thiobarbituric acid reactive substances
TEM	Transmission electron microscopy
TGF-β	Transforming growth factor β
TNF-α	Tumor necrosis factor alpha
TOS	Total oxidant status
TSH	Total native thiol
tBHP	tert-Butyl hydroperoxide
