# Supplementary material for: Pantothenic Acid Derivatives Modulate Oxidative Stress and Hepatic Fibrosis in Bile Duct Ligation-Induced Cholestatic Liver Injury
Source: Pathophysiology. 2026 May 13;33(2):32. doi: 10.3390/pathophysiology33020032 (PMC13214685; doi:10.3390/pathophysiology33020032)
Supplement: Supplementary file 1 [file pathophysiology-33-00032-s001.zip › pathophysiology-4204194-supplementary.pdf]

Pantothenic Acid Derivatives Modulate Oxidative Stress and Hepatic Fibrosis in Bile Duct Ligation–Induced Cholestatic Liver Injury

Dmitry S. Semenovich, Polina A. Abramicheva, Ljubava D. Zorova, Andrey V. Elchaninov, Maria A. Kozlova, David A. Areshidze, Nadezda V. Andrianova, Nina P. Kanunnikova, Andrey G. Moiseenok, Irina B. Pevzner, Egor Y. Plotnikov, and Dmitry B. Zorov

Supplementary materials

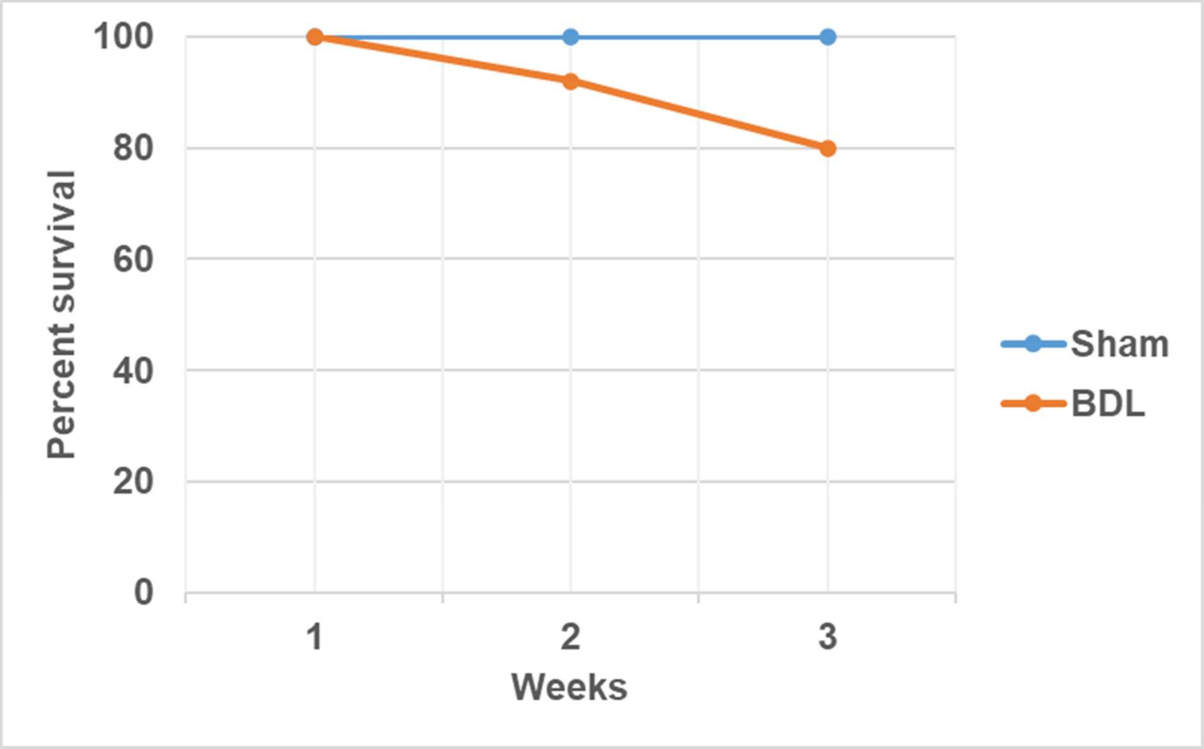

Figure S1. Survival dynamics of rats after common bile duct ligation (BDL) during the first three weeks after obstruction. Survival in sham-operated rats (n=5) was 100%. In rats with BDL (n=25), survival was 80% (week 3 of the experiment) — 5 rats out of 25 died. Two rats died due to non-specific postoperative complications, and three rats died due to progressive acute cholestasis and sepsis.

Note:

After 3 weeks of obstruction and until the end of the experiment (up to 6 weeks), rats were administered pantothenic acid derivates (panthenol, pantethine, and hopantenic acid) for treatment. Survival with these drugs in all groups was 100% (data not shown graphically).

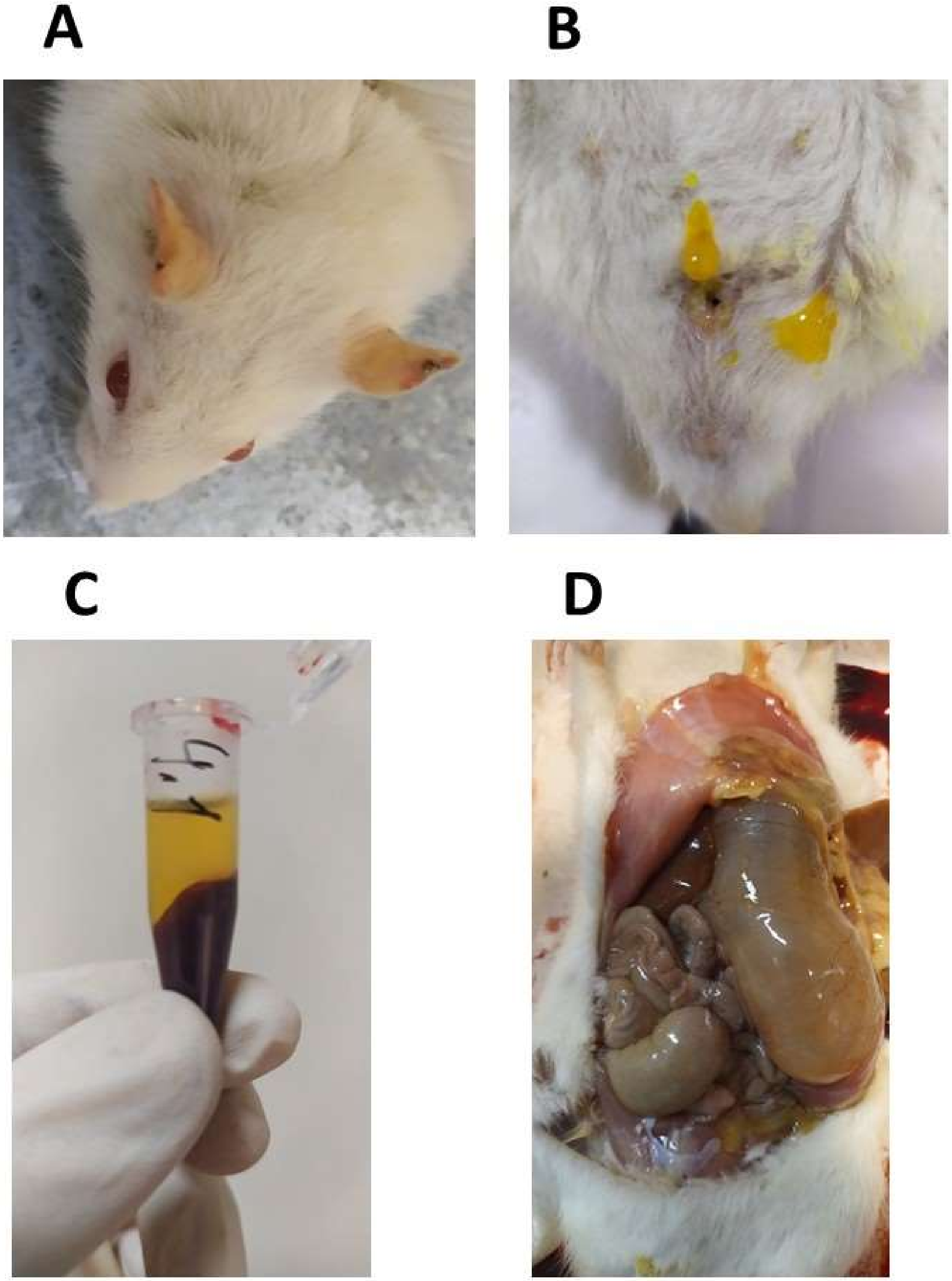

Figure S2. Symptoms of obstructive cholestasis in rats. (A) Jaundice. (B) Bilirubinuria (qualitatively confirmed by rapid urine test, Siemens Multistix, 10 SG, New York, USA). (C) Serum icterus. (D) Autopsy of a rat with BLB 6 weeks after obstruction. Note: The results of the biochemical analysis of rat blood serum are presented in Figure 2 of the manuscript (Section 3.2, Chapter 3 "Results").

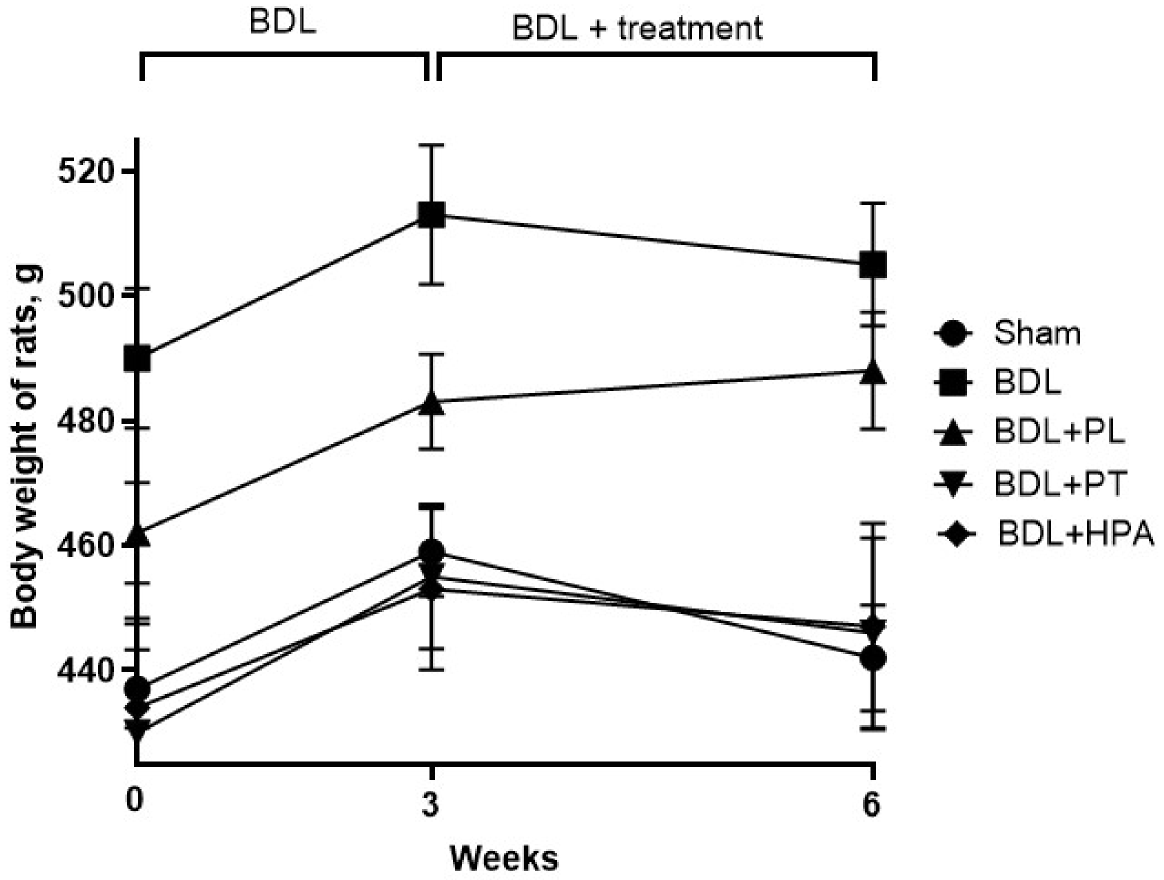

Figure S3. Dynamics of changes in the weight of sham-operated rats and rats with common bile duct ligation (BDL) before and after treatment with pantothenic acid derivates — panthenol (PL), pantethine (PT), hopantenic acid (HPA).

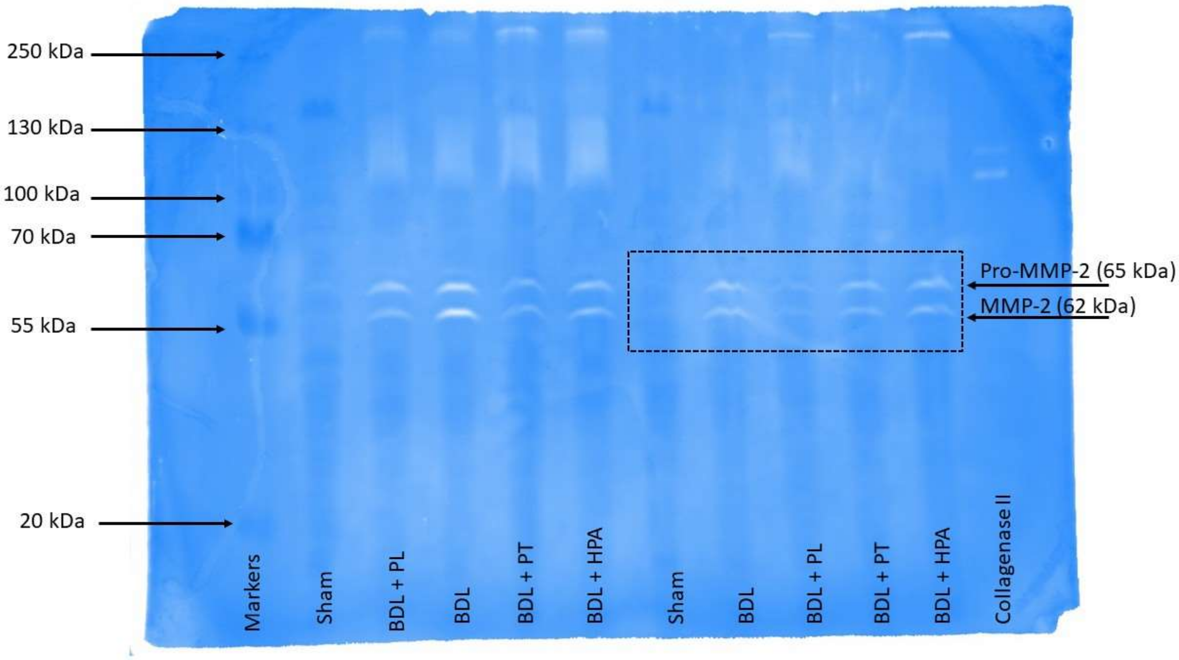

Figure S4. Gelatin zymography. Polyacrylamide gel with gelatin as a substrate. To assess MMP activity, the gel was stained with Coomassie R-250. Collagenase II was used as a positive control. The fragment presented in the manuscript was highlighted with a dotted frame.

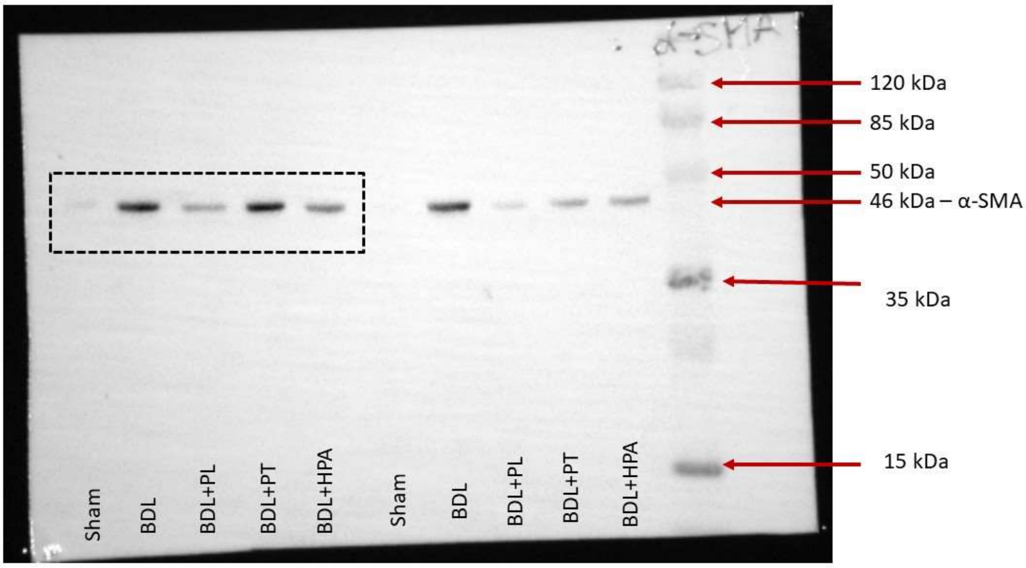

Figure S5. Evaluation of alpha-smooth muscle actin (α-SMA) expression by Western blotting. The image shows a developed PVDF membrane. The dotted frame highlights a fragment for representative illustration. The molecular weights of the marker proteins are indicated by arrows on the right.

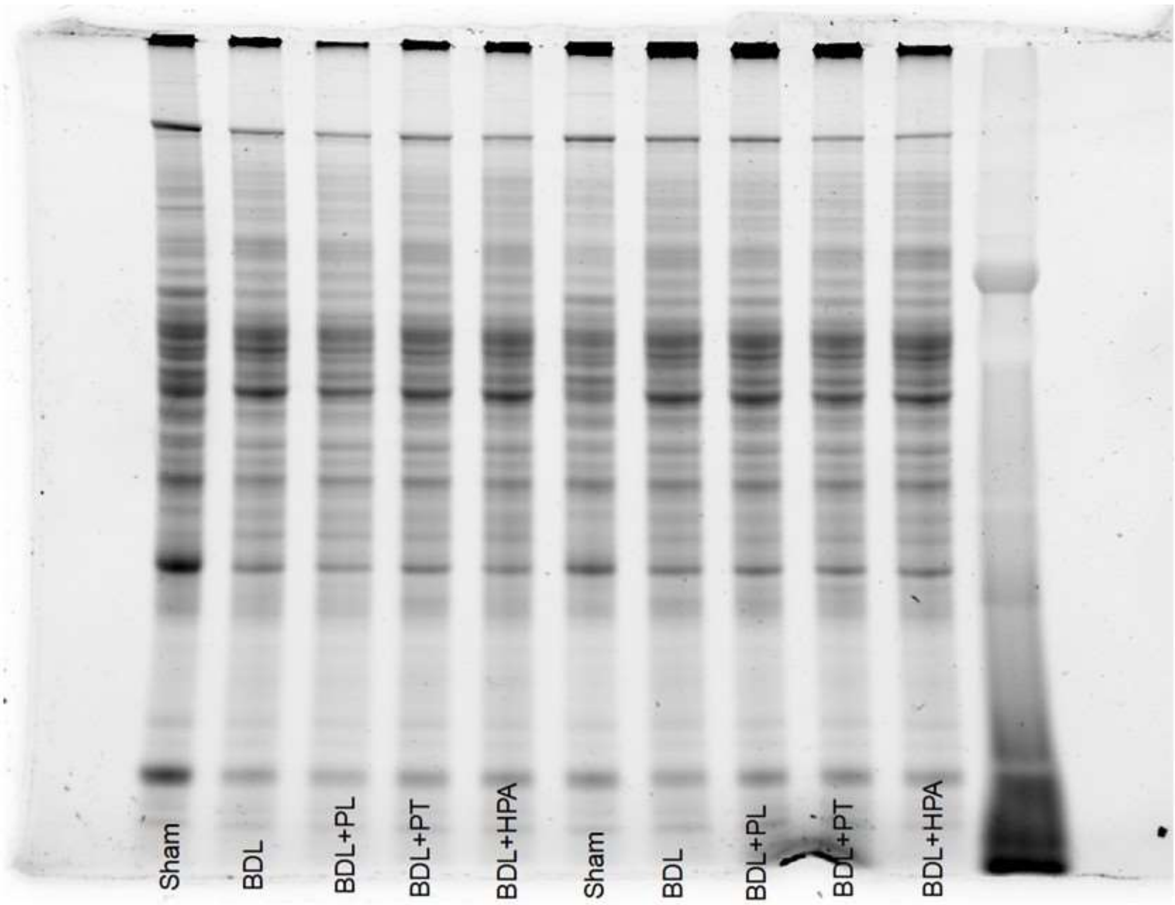

Figure S6. TGX Stain-Free Protein Visualization Gel. It was used to normalize the α-SMA signal.

Supplementary Table S1. Number of rats in each experimental group for specific protocols and methods.

**Methods and protocols**	**Experimental groups**				
	**Sham**	**BDL**	**BDL+PL**	**BDL+PT**	**BDL+HPA**
Blood biochemistry	5	5	5	5	5
Pathomorphology	5	5	5	5	5
Transmission electron microscopy	4	4	4	4	4
Hydroxyproline assay	5	5	5	5	5
Gelatin zymography (MMP activity assay)	4	4	3	4	4
Western blot (α-SMA)	2	4	4	4	3
RT-PCR (*Il6, Tnfa, Rplp0)*	4	5	5	5	5
Reduced glutathione and protein mix disulphides	5	5	5	5	5
Isolation of liver mitochondria	5	5	5	5	5
TBARS assay in mitochondria	5	5	5	5	5
SOD, GS, GR, GPx activity in cytosol fraction	5	5	5	5	5

Note:

The number of rats per group (n) may be less than 5 in the table due to technical limitations in implementing specific protocols and the presence of outliers in the data, as assessed using the Grubbs test.

Supplementary Table S2. Primer sequences used to estimate gene expression.

Protein	Gene name	Primer nucleotide sequence (5′-to 3′)	PCR product size, bp	Genbank accession number
Interleukin 6	*Il6*	forward AAGCCAGAGTCATTCAGAGCAA<br>reverse TTGGATGGTCTTGGTCCTTAGC	161	NM_012589.2
Tumor necrosis factor α	*Tnfa*	forward CTCCCTCTCATCAGTTCCATGG<br>reverse TGGTATGAAATGGCAAATCGGC	296	NM_012675.3
60S acidic ribosomal protein P0	*Rplp0*	forward CACAGTACCTGCTCAGAACAC<br>reverse ACCTTGTCTCCAGTCTTTATCAG	138	NM_022402.2

Supplementary Table S3. Criteria for the Ishak fibrosis scoring system [54].

Scores	Definition
0	No fibrosis
1	Fibrous expansion of some portal areas, with or without short fibrous septa
2	Fibrous expansion of most portal areas, with or without short fibrous septa
3	Fibrous expansion of most portal areas with occasional portal to portal bridging
4	Fibrous expansion of portal areas with marked bridging (portal to portal as well as portal to central)
5	Marked bridging (portal–portal and/or portal–central) with occasional nodules (incomplete cirrhosis)
6	Cirrhosis

Supplementary Table S4. Criteria for the METAVIR fibrosis staging system [55].

Stage	Definition
F0	No fibrosis
F1	Portal fibrosis without septa
F2	Portal fibrosis with few septa
F3	Numerous septa without cirrhosis
F4	Cirrhosis
